# Structural adaptability and hy­dro­gen bonding in a dissymmetric pyrimidine thio­ether ligand

**DOI:** 10.1107/S205322962500823X

**Published:** 2025-10-06

**Authors:** Kaycee Anoliefo, Kaitlyn Brown, Lana K. Hiscock, Paul D. Boyle, Louise N. Dawe

**Affiliations:** a Department of Chemistry and Biochemistry, Wilfrid Laurier University, 75 University Ave. W., Waterloo, Ontario, N2L 3C5, Canada; b Department of Chemistry, York University, 4700 Keele Street, Toronto, Ontario, M3J 1P3, Canada; c Department of Chemistry X-ray Facility, University of Western Ontario, 1151 Richmond Street North, London, Ontario, N6A 5B7, Canada; University of North Texas at Dallas, USA

**Keywords:** crystal structure, pyrimidine thio­ether ligand, hy­dro­gen bonding, coordination chemistry, Hirshfeld surface analysis, structure adaptability, disymmetric

## Abstract

Dissymmetric ligands have garnered inter­est due to their ability to simultaneously coordinate to multiple different acceptors. Herein, we report the syn­thesis of a dissymmetric thio­ether *N*,*N*′-bidentate ligand, namely, 2-[(6-chloro­pyrimidin-4-yl)sulfan­yl]pyrimidine-4,6-di­amine (C_8_H_7_ClN_6_S, **L1**), along with its hydrated form (C_8_H_7_ClN_6_S·H_2_O). In addition, we describe the structure of a nitrate salt of the protonated ligand {4,6-di­amino-2-[(6-chloro­pyrimidin-4-yl)sulfan­yl]pyrimidin-1-ium nitrate, C_8_H_8_ClN_6_S^+^·NO_3_^−^} and a cobalt(II) com­plex of **L1** (di­chlorido­{2-[(6-chloro­pyrimidin-4-yl-κ*N*^3^)sulfan­yl]pyrimidine-4,6-di­amine-κ*N*^3^}cobalt(II), [CoCl_2_(C_8_H_7_ClN_6_S)]). The structures of all four com­pounds were determined by single-crystal X-ray diffraction and Hirshfeld surface analyses were performed. These analyses reveal unengaged hy­dro­gen-bond donors and acceptors in both the neutral ligand and its water solvate, while protonation or metal coordination induces a conformational change that en­ables full engagement of hy­dro­gen-bond donors. These structural insights have implications for the mol­ecular design of ligands in ion-sensing applications.

## Abstract 2

A dissymmetric pyrimidine thio­ether *N*,*N*′-bidentate ligand was synthesized along with its hydrated form, nitrate salt and a cobalt(II) com­plex. All four structures were characterized by single-crystal X-ray diffraction and Hirshfeld surface analysis to evaluate their potential for ion sensing *via* hy­dro­gen-bonding inter­actions.

## Introduction

Our group is inter­ested in the structure, properties and appli­cations of multifunctional ligands capable of coordinating to metal cations and participating in inter­molecular inter­actions with anions. We are particularly motivated by a knowledge gap related to the directionality of weak inter­molecular forces, which challenges the rational design of functional coordination com­plexes (Molina *et al.*, 2017 ▸; Chakrabarty *et al.*, 2011 ▸; Desiraju, 2007 ▸). Among the systems of inter­est are dissymmetric ligands, which have garnered attention for their capacity to simultaneously coordinate different metal cations and to mediate a range of noncovalent inter­actions (Liu *et al.*, 2018 ▸; Yang *et al.*, 2016 ▸). These ligands, and their resulting coordination com­plexes, have demonstrated applications across catalysis, magnetism and the design of novel supra­molecular architectures (Adilkhanova *et al.*, 2023 ▸; Worrell *et al.*, 2023 ▸; Xu *et al.*, 2022 ▸).

**Figure 1:**
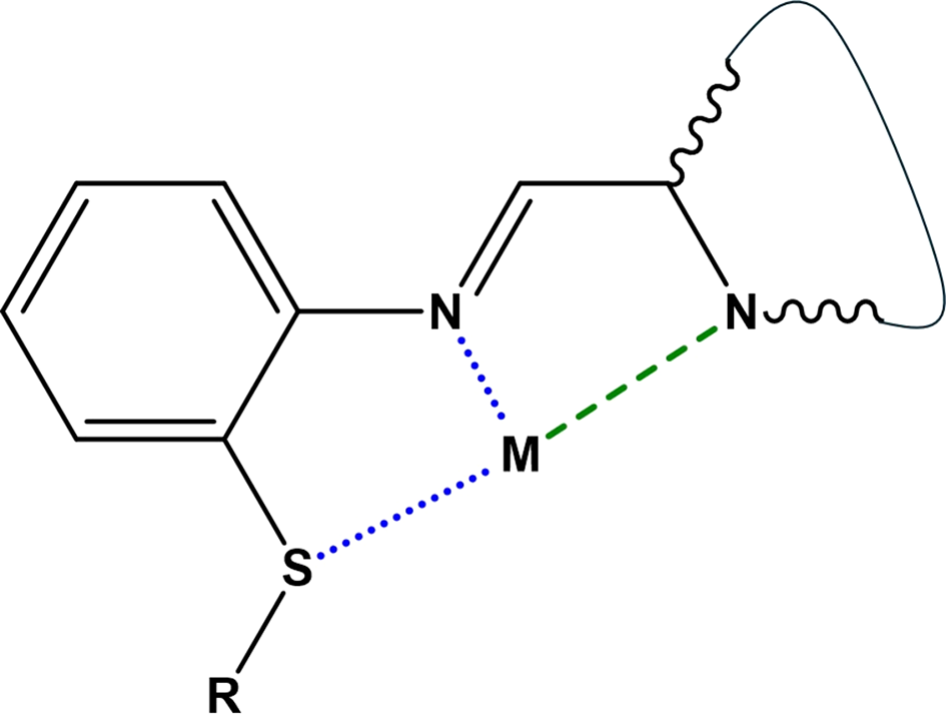
Previously reported *N*,*N*′,*S*-donor ligands capable of *N*,*S*-bidentate (blue) or *N*,*N*′,*S*-tridentate (blue and green) metal coordination [based on the work of Dehghani-Firouzabadi *et al.* (2016 ▸, 2017 ▸, 2020 ▸)]. The presence of coligands at the metal site, M, results in four-, five- or six-coordinated com­plexes.

Recent inter­est has focused on dissymmetrical Schiff base ligands which can support various metal-to-ligand coordination modes and stoichiometries (Costes *et al.*, 2020 ▸; Liu *et al.*, 2018 ▸; Dehghani-Firouzabadi *et al.*, 2016 ▸). Examples include those with *N*,*N*′,*S*-donor sets, which can act as *N*,*S*-bidentate or *N*,*N*′,*S*-tridentate ligands and form stable four-, five- or six-coordinated com­plexes (Dehghani-Firouzabadi *et al.*, 2017 ▸, 2020 ▸) (Fig. 1 ▸).

Strategic ligand design in coordination chemistry can be used to promote direct inter­action between cationic metal centres and target anions. Such inter­actions occur through primary-sphere coordination, wherein the anion occupies a metal coordination site (Mercer & Loeb, 2010 ▸). However, achieving selectivity and stability under com­petitive conditions, especially in polar solvents, often requires second-sphere coordination. In this approach, the anion binds to a ligand that is already coordinated to a metal centre, *via* hy­dro­gen bonding or electrostatic inter­actions (Hiscock *et al.*, 2019 ▸; Moyaert *et al.*, 2018 ▸; Mercer & Loeb, 2010 ▸). These inter­actions can be perturbed or promoted by solvent effects, as solvent mol­ecules may act as hy­dro­gen-bond acceptors or coordinate directly to the metal centre, thereby altering the binding environment (Zhao *et al.*, 2019 ▸; Robertson *et al.*, 2017 ▸).

**Figure 2:**
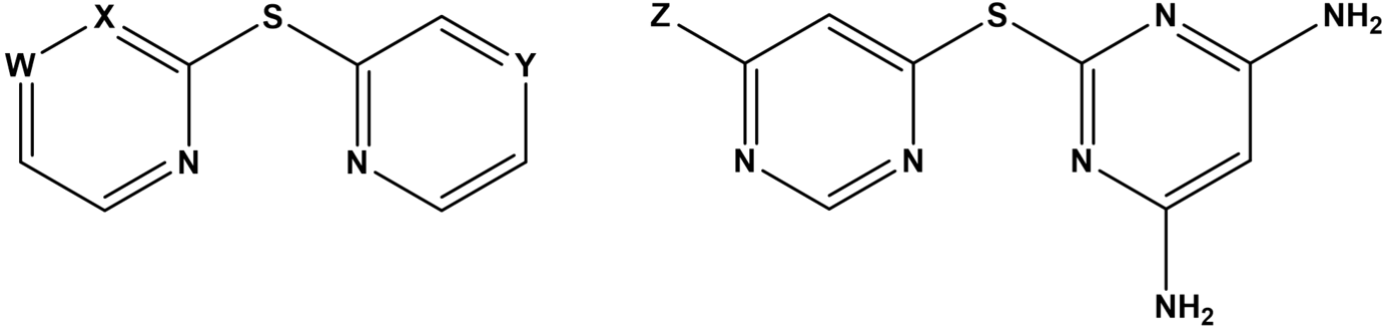
(Left) Previously reported thio­ether ligands with W = X = Y = CH (Teles *et al.*, 2006 ▸); X = Y = N, W = CH; and W = Y = N, X = CH (Fresta *et al.*, 2022 ▸). (Right) *N*,*N*′-Bidentate ligands derived from 4,6-di­chloro­py­rimi­dine, with Z = N(CH_3_)_2_ (Moyaert *et al.*, 2017 ▸) or Cl (**L1**; this work).

Second-sphere inter­actions are especially relevant to supra­molecular assembly strategies. For example, Teles *et al.* (2006 ▸) demonstrated the use of thio­ether-based *N*,*N*′-bidentate spacer ligands in the self-assembly of supra­molecular arrays, relying on directional noncovalent inter­actions (Fig. 2 ▸). Similarly, the design of luminescent materials often exploits these principles (Pashaei *et al.*, 2019 ▸); for example, Fresta *et al.* (2022 ▸) reported red-emitting copper(I) com­plexes incorporating pyrimidinyl ligands (Fig. 2 ▸) for use in white light-emitting electrochemical cells, where secondary inter­actions contribute to emissive properties and mol­ecular packing.

In the present work, we examine a dissymmetric thio­ether *N*,*N*′-bidentate ligand derived from 4,6-dichlorpyrimidine, which reflects this design logic, and builds on our previously reported work (Moyaert *et al.*, 2017 ▸). Herein, we report the synthesis and solid-state characterization of 2-[(6-chloro­pyri­midin-4-yl)sulfan­yl]pyrimidine-4,6-di­amine (**L1**), its hydrated form (**L1·H_2_O**), its protonated nitrate salt (**[L1+H][NO_3_]**) and its cobalt(II) com­plex (**L1CoCl_2_**). Single-crystal X-ray dif­frac­tion studies, supported by Hirshfeld surface analysis, were undertaken to assess the primary and secondary coordination features of these structures. These results lay the groundwork for future studies on ligand modification and com­plexation, including the introduction of additional functionality at the chloro-substituted pyrimidine ring to support extended coordination motifs.

## Experimental

### General procedures

The ^1^H NMR NMR spectrum was recorded on an Agilent Technologies Varian Unity Inova 400 MHz NMR spec­trometer. Chemical shifts are reported in δ scale using the residual ^1^H solvent peak (DMSO-*d*_6_, δ = 2.50 ppm) as reference. 4,6-Di­chloro­pyrimidine (TCI) and 4,6-di­amino­py­rimi­dine-2-­thiol (Merck) were used as purchased. All other reagents and starting materials were purchased from Sigma–Aldrich and used as purchased. Melting points were determined on a Mel-Temp electrothermal melting-point apparatus and are uncorrected. Single crystals were selected and col­lected on a Bruker APEXII CCD diffractometer at Western University, London, ON, Canada. Crystals were kept at 110 (2) K during data collection. Using *OLEX2* (Dolomanov *et al.*, 2009 ▸), the structures were solved with the *SHELXT* (Sheldrick, 2015*a* ▸) structure solution program using direct methods and refined with the *SHELXL* (Sheldrick, 2015*b* ▸) refinement package using least-squares minimization.

### Refinement

Crystal data, data collection and structure refinement details are summarized in Table 1 ▸. All H atoms, except where noted otherwise, were placed geometrically (C—H = 0.95 Å) and refined using a riding model, with *U*_iso_(H) = 1.2*U*_eq_ of the carrier atom. All non-H atoms were refined anisotropically. For **L1**, **L1·H_2_O** and **[L1+H][NO_3_]**, amine H atoms (bound to N5 and N6 in all structures, and additionally to N11 and N12 in **[L1+H][NO_3_]**) were located in difference maps, refined positionally with similarity restraints (*SHELXL* SADI) and treated isotropically, while in **L1CoCl_2_**, these H atoms were refined with an isotropic displacement fixed at 1.5 times that of the carrier atom. For **L1·H_2_O**, the H atoms on the O atom (*i.e.* water atom O1) were treated similarly. In **[L1+H][NO_3_]**, *Z*′ = 2, and one disordered nitrate anion was present with occupancies of 0.672 (3) and 0.328 (3). The O atoms of this group were constrained to have identical anisotropic displacements (*SHELXL* EADP). For **L1CoCl_2_**, a minor second twin domain (BASF < 8%) was identified. Including this domain (*SHELX* HKLF5) resulted in higher refinement statistics and a model that did not converge satisfactorily. Therefore, the second domain was excluded from the final refinement, which was performed using the single-domain HKLF4 reflection file.

### Synthesis of L1

4,6-Di­chloro­pyrimidine (2.00 g, 13.4 mmol, 1.00 equiv.) was added to a 250 ml round-bottomed flask con­taining 100 ml of ethanol. Tri­ethyl­amine (1.87 ml, 13.5 mmol, 1.00 equiv.) was added *via* syringe and the mixture was stirred at room tem­per­a­ture for 15 min, resulting in a clear colourless solution. Subsequently, 20 ml of dimethylformamide (DMF) and 1.908 g of 4,6-di­amino­pyrimidine-2-thiol (13.5 mmol, 1.00 equiv.) were added, yielding an opaque white solution. The reaction mixture was heated under reflux for 48 h, resulting in a clear yellow solution with a white precipitate upon cooling to room tem­per­a­ture. Deionized water (150 ml) was added, dissolving the precipitate. The solution was transferred to a 500 ml round-bottomed flask and con­cen­trated by rotary evaporation to approximately 100 ml. The residue was extracted three times with di­chloro­methane using a 500 ml separatory funnel. The combined organic layers were dried over anhydrous MgSO_4_, filtered by gravity and concentrated by rotary evaporation to afford a dark red–yellow oil. After cooling to room tem­per­a­ture, 50 ml of cold deionized water were added and the mixture was stirred in an ice bath for 24 h. The resulting white solid was collected by suction filtration and dried to give the product as a white solid (yield: 1.365 g, 55.8%). ^1^H NMR (400 MHz, DMSO): δ 8.79 (*s*, 1H), 8.46 (*s*, 1H), 6.45 (*s*, 4H), 5.26 (*s*, 1H). IR (ATR, cm^−1^): 3610, 3441, 3309 (NH_2_); 751 (C—S); 1637 (C=N); 806 (C—Cl). HRMS (TOF) *m*/*z*: [*M* + H] + Calcd for C_8_H_7_ClN_6_S 255.02142; found 255.02171. The reaction scheme is presented in Scheme 1.
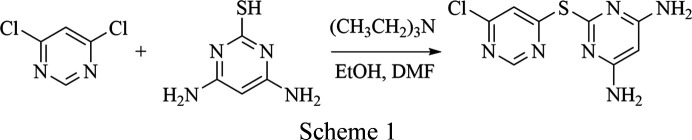


### Synthesis of L1·H_2_O and [L1+H][NO_3_]

Unsuccessful attempts to synthesize Lewis acid/base com­plexes using a methodology consistent with that employed for **L1CoCl_2_** (*vide infra*), but with a mixed solvent system of aceto­­nitrile, ethyl acetate and water, resulted instead in the formation of crystals of the hydrate of **L1**, **L1·H_2_O**, when MnSO_4_·4H_2_O was used, and the nitrate salt of the protonated ligand, **[L1+H][NO_3_]**, when Ga(NO_3_)_3_·H_2_O was used. In the presence of water and protic solvents, protonation or solvation of the ligand were favoured over cation coordination, and metal com­plexes were not isolable.

### Synthesis of L1CoCl_2_

CoCl_2_·6H_2_O (0.187 g, 0.785 mmol, 2 equiv.) and **L1** (0.100 g, 0.393 mmol, 1 equiv.) were each dissolved in 10 ml of a 1:1 (*v*/*v*) ethanol/methanol mixture. The solution of CoCl_2_·6H_2_O (clear dark purple) was added dropwise to the solution of **L1**, yielding a clear dark-blue solution. The mixture was heated to approximately 60 °C and stirred for 15 min. The solution was then filtered by gravity into a small vial and left undisturbed to crystallize. Blue X-ray-quality crystals of **L1CoCl_2_** were ob­tained after two weeks.

## Results and discussion

**Figure 3:**
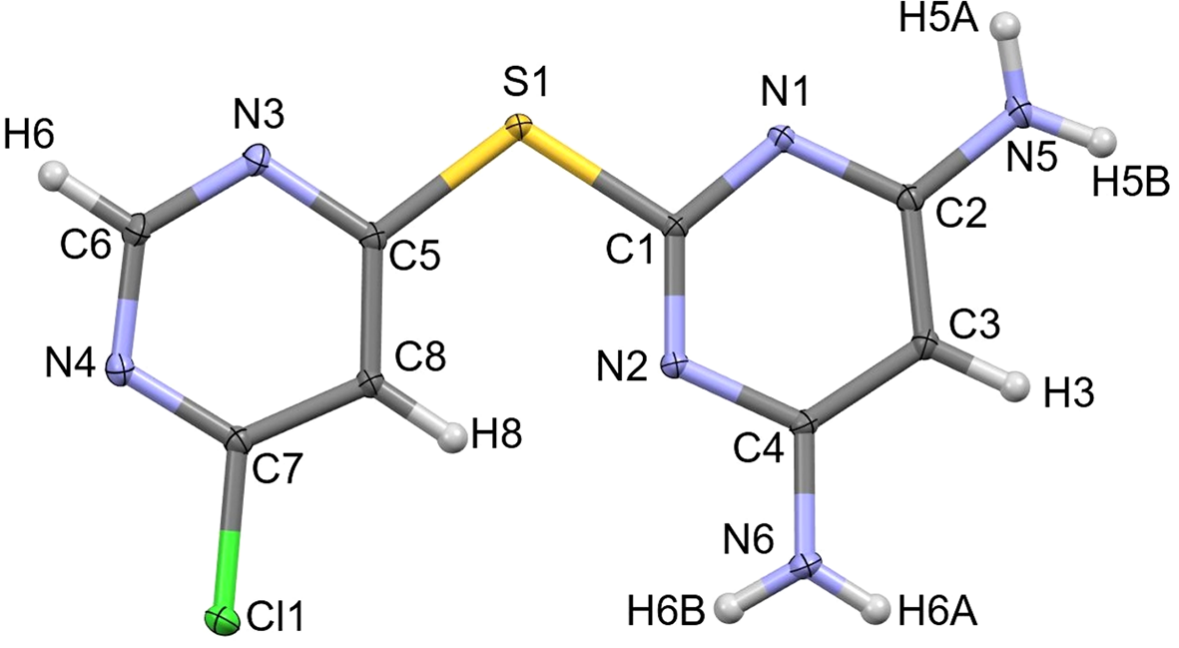
The asymmetric unit of **L1**, drawn with 50% probability displacement ellipsoids for the non-H atoms.

**L1** crystallized in the noncentrosymmetric ortho­rhom­bic space group *Pna*2_1_ with one formula unit, C_8_H_7_ClN_6_S, in the asymmetric unit (Fig. 3 ▸). The mol­ecule adopts a nearly planar orientation; considering the plane formed by the two pyrimidine rings and atom S1, the root-mean-square deviation (RMSD) is 0.046 Å. The chloro-substituted ring is oriented such that C8—H8 points toward amine-functionalized pyrimidyl atom N2, but no intra­molecular hy­dro­gen bond is formed (∠C8—H8⋯N2 = 126°).

**Figure 4:**
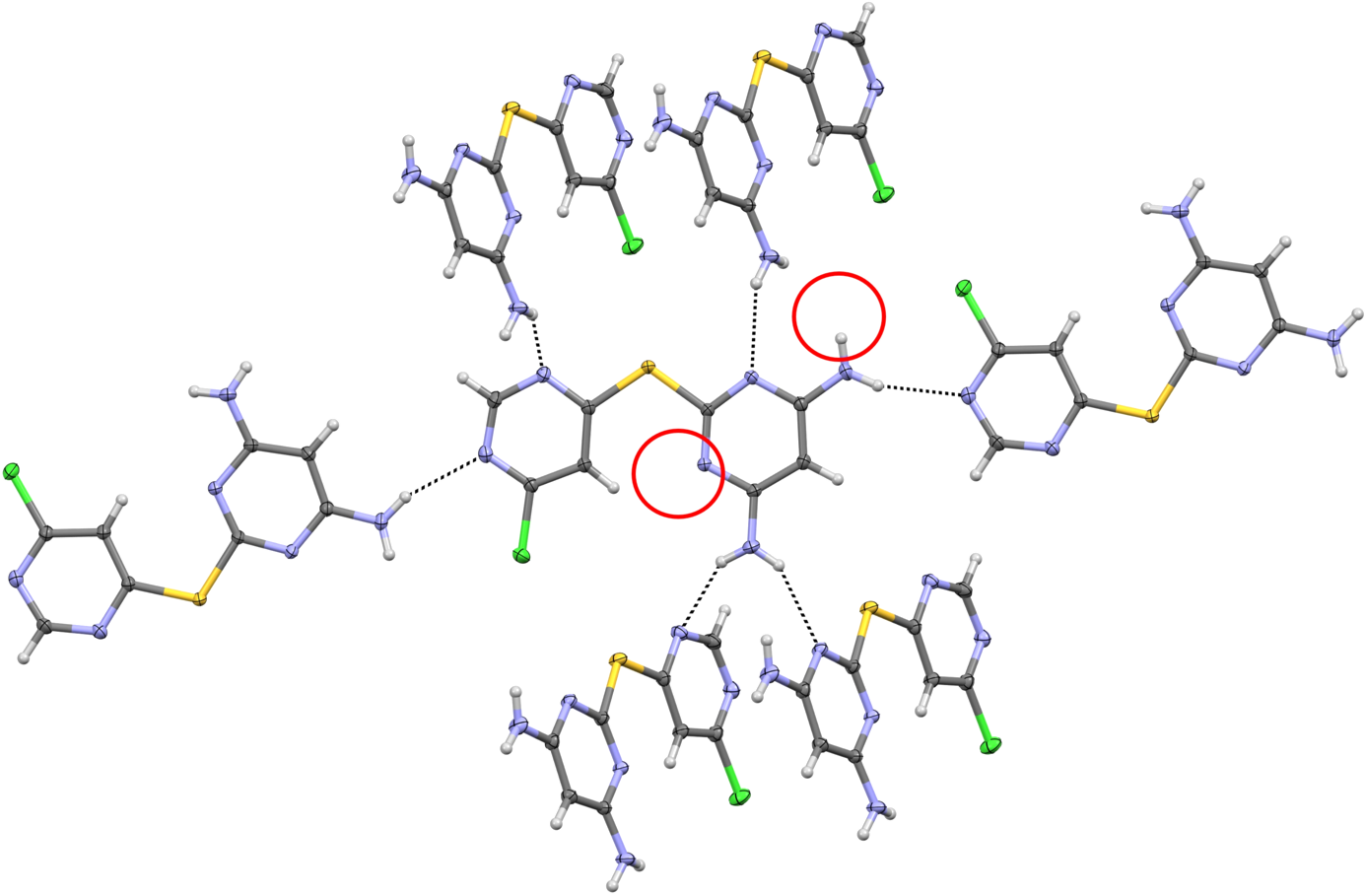
Extended packing diagram for **L1**, drawn with 50% probability displacement ellipsoids for the non-H atoms. Hydrogen bonds are represented as dashed lines. Unengaged hy­dro­gen-bond donors and acceptors in the asymmetric unit are indicated by red circles.

Although the number of hy­dro­gen-bond donors and acceptors is balanced in the asymmetric unit, examination of the packing motif reveals that neither pyrimidinyl atom N2 nor the amine hy­dro­gen-donor N5—H5*A* group participates in hy­dro­gen bonding (Fig. 4 ▸). In contrast, simple inter­molecular hy­dro­gen-bond graph-set motifs *C*(6), *C*(8) and *C*(10) account for inter­actions involving the remaining donors and acceptors (Table 2 ▸). We, and others, have previously reported challenges related to the self-com­plementarity of mol­ecules designed for anion sensing, namely, that such sensors may preferentially engage in hy­dro­gen bonding with one another unless some acceptor sites are occupied through coordination to metal cations (Hiscock *et al.*, 2019 ▸; Mercer & Loeb, 2010 ▸; Qureshi *et al.*, 2016 ▸). Notably, the unengaged hy­dro­gen-bond donor in **L1** suggests potential for selective anion inter­actions, even in the absence of cation coordination to the available hy­dro­gen-bond acceptors.

**Figure 5:**
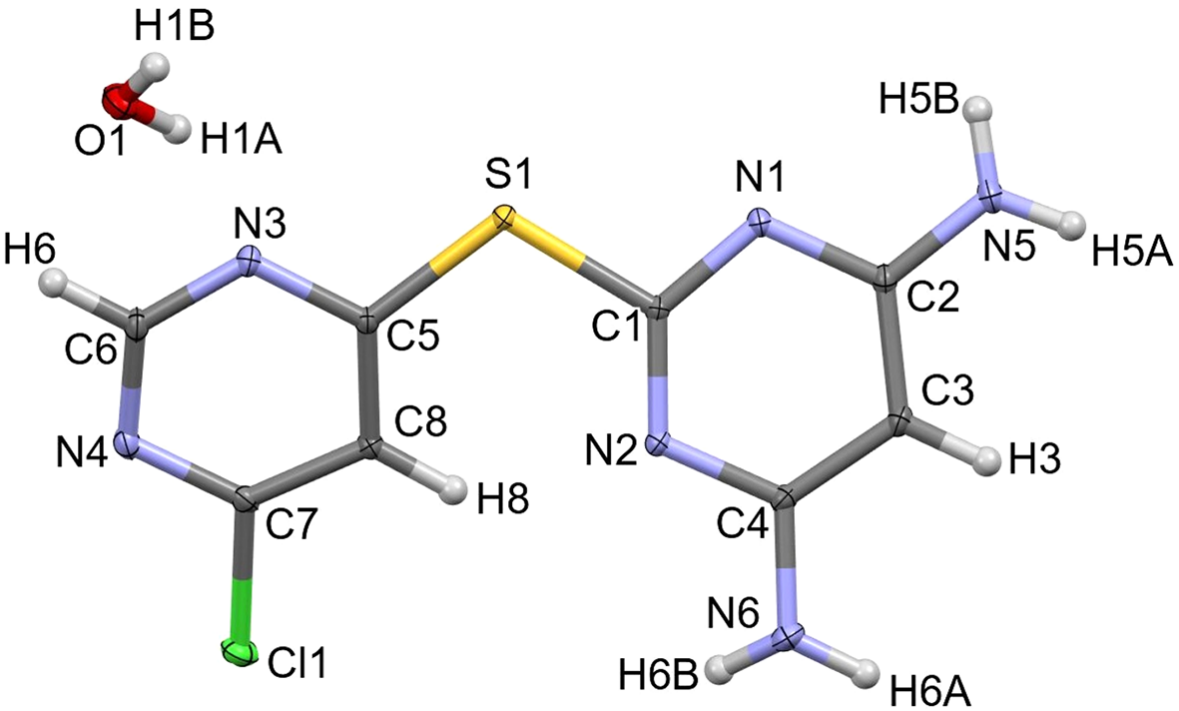
The asymmetric unit of **L1·H_2_O**, drawn with 50% probability displacement ellipsoids for the non-H atoms.

**L1·H_2_O** crystallized in the centrosymmetric monoclinic space group *P*2_1_/*n* with one formula unit, C_8_H_7_ClN_6_S·H_2_O, in the asymmetric unit (Fig. 5 ▸). Similar to the unsolvated structure of **L1** (*vide supra*), the chloro-substituted ring is again oriented such that C8—H8 points toward amine-functionalized pyrimidyl atom N2, but no intra­molecular hy­dro­gen bond is formed (∠C8—H8⋯N2 = 125°). In contrast, **L1** now adopts a twisted conformation, with a dihedral angle of 25.37 (2)° between the planes of the two pyrimidine rings.

**Figure 6:**
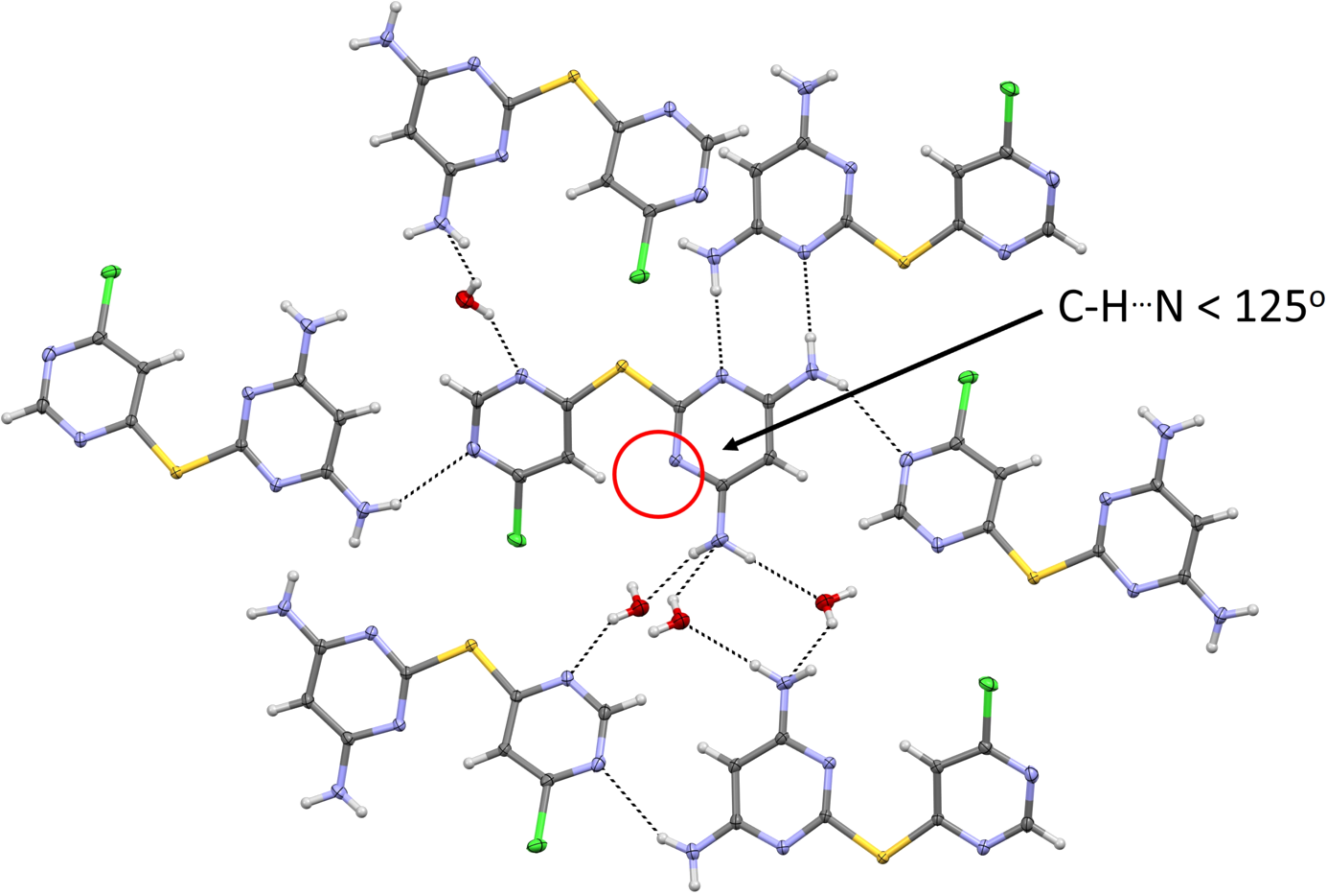
Extended packing diagram for **L1·H_2_O**, drawn with 50% probability displacement ellipsoids for the non-H atoms. Hydrogen bonds are represented as dashed lines. The unengaged hy­dro­gen-bond acceptor in the asymmetric unit is indicated by a red circle.

In the structure of **L1·H_2_O**, all amine protons are engaged in inter­molecular inter­actions, either with pyrimidinyl N-atom acceptors from neighbouring **L1** mol­ecules or with the O atom of the lattice water mol­ecule. However, as in the unsolvated structure, pyrimidinyl atom N2 again does not participate in hy­dro­gen bonding (Fig. 6 ▸). Accounting for the amine donors, the primary inter­molecular hy­dro­gen-bonding network is described by the graph-set motifs 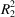
(8), *C*(10) and *D*(2) (Table 2 ▸). The continued absence of hy­dro­gen bonding at the N2 acceptor site remains notable, especially given the pres­ence of the lattice water mol­ecule.

**Figure 7:**
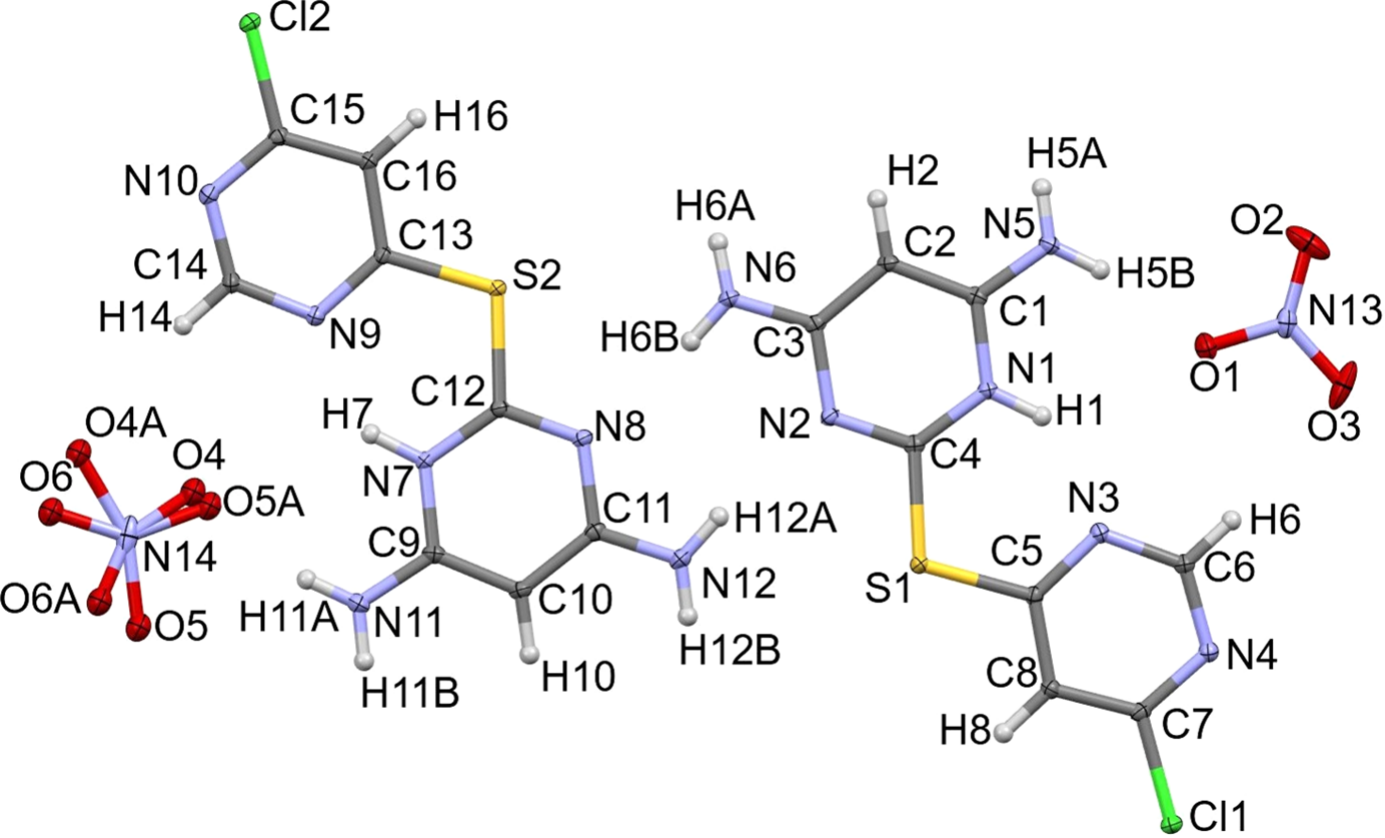
The asymmetric unit of **[L1+H][NO_3_]**, drawn with 50% probability displacement ellipsoids for the non-H atoms.

The **[L1+H][NO_3_]** salt crystallized in the centrosymmetric monoclinic space group *P*2_1_/*c* with two formula units of C_8_H_8_ClN_6_S·NO_3_ in the asymmetric unit (Fig. 7 ▸). One nitrate anion is ordered, while the second is disordered over two refined orientations, with occupancies of 0.672 (3) and 0.328 (3), respectively. Unlike **L1** and its water solvate, the amine-functionalized *endo* pyrimidinyl atom (N1 and N7) is now protonated and engaged in intra­molecular hy­dro­gen bonding with the chlorine-bearing pyrimidinyl ring in both formula units of **[L1+H][NO_3_]** (both ∠N1—H1⋯N3 and ∠N7—H7⋯N9 = 142°).

**Figure 8:**
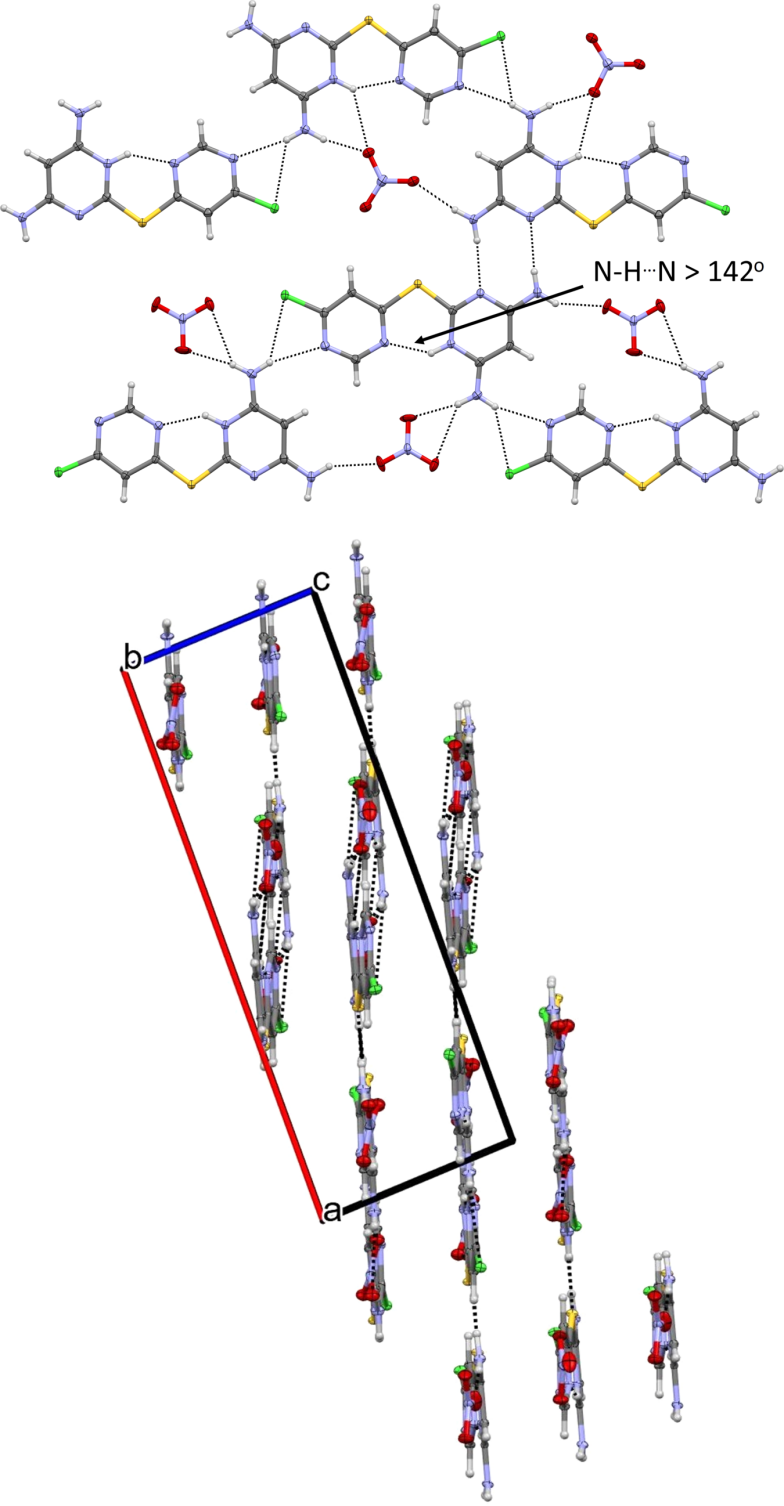
Extended packing diagrams for **[L1+H][NO_3_]**, drawn with 50% probability displacement ellipsoids for the non-H atoms. Hydrogen bonds are represented as dashed lines. The minor disorder com­ponent has been omitted for clarity.

In the structure of **[L1+H][NO_3_]**, all strong hy­dro­gen-bond donors and acceptors participate in intra- or inter­molecular inter­actions (Fig. 8 ▸). Accounting for all N—H donors, the primary inter­molecular hy­dro­gen-bonding network is de­scribed by the graph-set motifs 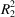
(8), *C*(10), *S*(6) and *D*(2) (Table 2 ▸). While included in Fig. 8 ▸, N—H⋯Cl inter­actions are weak, with bond angles less than 135°, and are therefore omitted from Table 2 ▸.

Considering all atoms in the asymmetric unit, the mol­ecules adopt a nearly planar orientation, with an RMSD of 0.161 Å. Packing analysis shows that the mol­ecular planes are separated by 3.1496 (16) Å and shifted by 2.349 (2) Å. Notably, **L1** is now oriented to form a coordination pocket defined by N1—C4—S1—C5—N3 (and equivalent atoms in the second formula unit); however, protonation of N1 (and N7) renders both N1 and N3 (or N7 and N9) inaccessible for inter­molecular self-com­plementary hy­dro­gen bonding, and instead facilitates amine–anion hy­dro­gen bonding.

**Figure 9:**
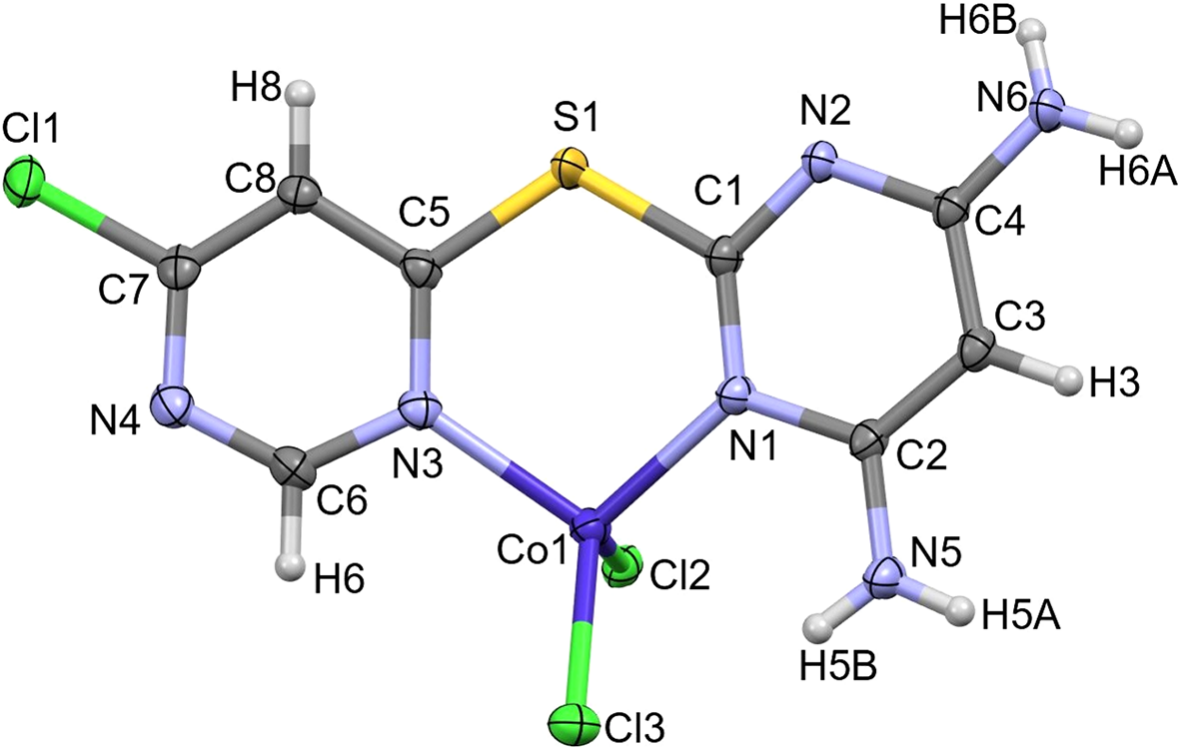
The asymmetric unit for **L1CoCl_2_**, drawn with 50% probability displacement ellipsoids for the non-H atoms.

The com­plex **L1CoCl_2_** also crystallized in the centrosymmetric monoclinic space group *P*2_1_/*c*, but with one C_8_H_7_ClN_5_SCoCl_2_ formula unit in the asymmetric unit (Fig. 9 ▸). This com­plex adopts a conformation similar to that of the **[L1+H][NO_3_]** salt, however, the coordination pocket defined by N1—C1—S1—C5—N3 is now bound to Co1 in a bidentate manner, forming a six-membered chelate ring.

The non-H atoms of **L1** adopt a nearly planar arrangement (RMSD = 0.104 Å), while the Co^II^ ion is puckered out of this plane, lying 0.6828 (19) Å below it. The Co1 atom adopts an approximately tetra­hedral geometry, with bond angles ranging from 99.89 (13) to 115.50 (10)°, and its charge is balanced by coordination to two chloride ions. The Co—Cl bond lengths are 2.2552 (13) and 2.2190 (13) Å, while the Co—N bond lengths are 2.013 (3) and 2.021 (3) Å.

A search of the Cambridge Structural Database (CSD, Version 5.46 with February 2025 updates; Groom *et al.*, 2016 ▸) using *ConQuest* (Bruno *et al.*, 2002 ▸) was conducted for similar systems; specifically, four-coordinated Co com­plexes with two chloride ligands and two N-atom donors forming a six-mem­bered chelate ring. This search yielded 155 Co—N and Co—Cl observations, with mean Co—Cl and Co—N bond lengths of 2.25 (4) and 2.02 (4) Å, respectively. The values observed for **L1CoCl_2_** are in excellent agreement with these literature/database-reported results.

**Figure 10:**
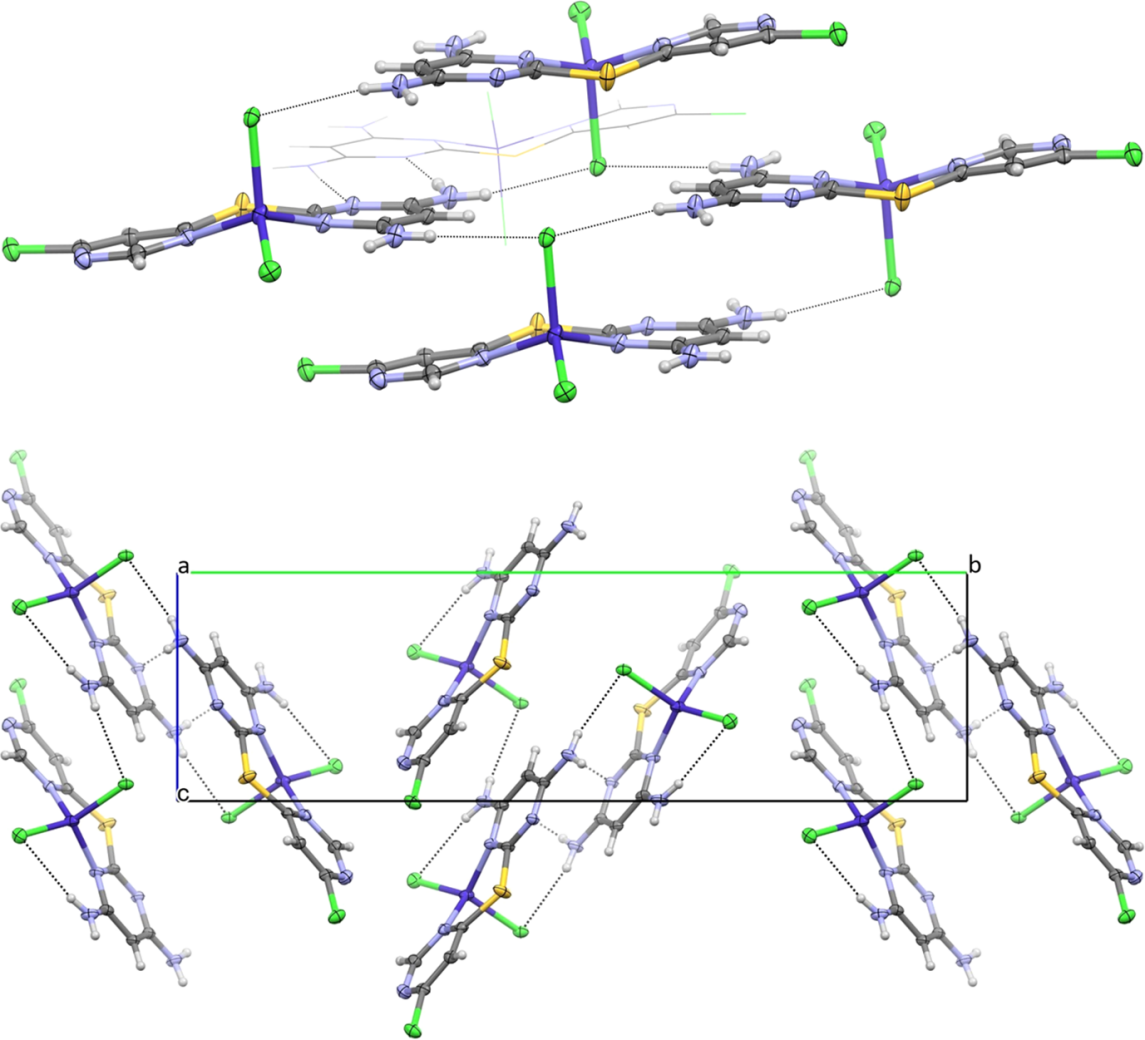
Extended packing diagrams for **L1CoCl_2_**, drawn with 50% displacement ellipsoids, except for one mol­ecule forming a dimer in the upper panel with wireframe representation for clarity. Hydrogen bonds are represented as dashed lines.

In the structure of **L1CoCl_2_**, all N—H donors participate in hy­dro­gen bonding, and the primary resulting network is de­scribed by the graph-set motifs 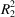
(8), *C*(10) and *S*(6) (Table 2 ▸). Self-com­plementary rings formed *via* N6—H6*B*⋯N2^xii^ [sym­metry code: (xii) −*x* + 2, −*y* + 1, −*z* + 2] generate dimers, which further engage in N5—H5*A*⋯Cl2^x^ [symmetry code: (x) *x*, *y*, *z* + 1] inter­actions with adjacent layers. This results in an 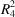
(16) graph-set motif, shown in the upper panel of Fig. 10 ▸. The layers are staggered through application of the 2_1_-screw axis (Fig. 10 ▸). Notably, neither pyrimidinyl atom N4 nor adjacent atom Cl1 participates in any significant inter­molecular inter­actions.

Hirshfeld surface analysis (Spackman & Jayatilaka, 2009 ▸) was performed using *CrystalExplorer17* (Spackman *et al.*, 2021 ▸). Examination of the Hirshfeld surfaces clearly shows a 180° bond rotation about the C—S bond to the chloro-sub­sti­tuted pyrimidine ring in the fully hy­dro­gen-bond-satisfied structure (*i.e.* the nitrate salt), aligning this conformation with that observed for the cobalt com­plex. In contrast, structures in which the ligand’s hy­dro­gen-bonding capacity is not fully utilized, and where no inter­nal hy­dro­gen bond is present, exhibit the opposite conformation of the chloropyrimidine ring. This difference is consistent with steric considerations: in the nitrate salt, the amine-functionalized pyrimidine ring is protonated, which would otherwise result in a steric clash with the proton on the chloro-substituted ring.

**Figure 11:**
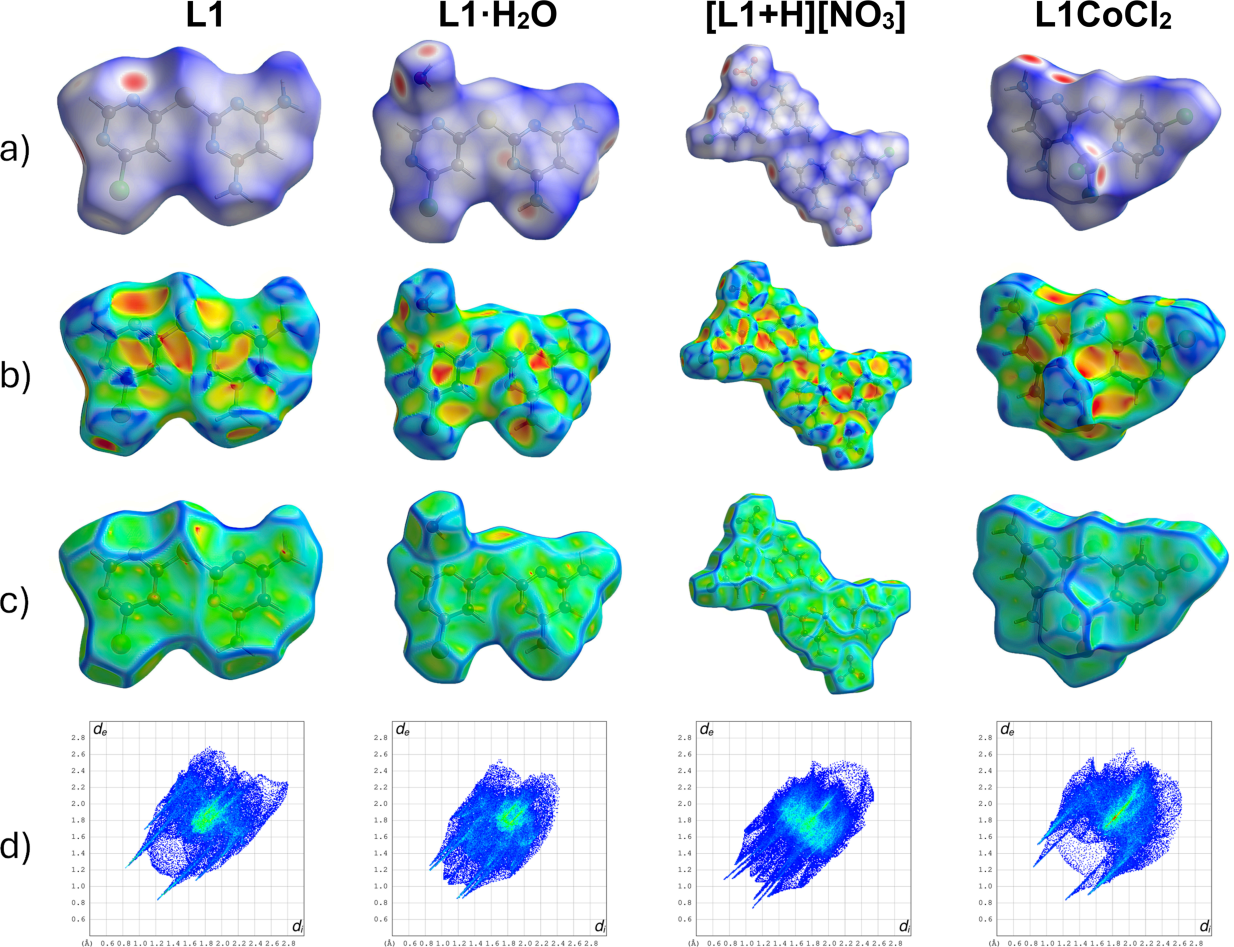
Hirshfield surfaces mapped with (*a*) *d*_norm_ ranging from −0.448 (red) to 1.47 (blue), (*b*) shape index, mapped from 1.0 (concave, red) through 0.0 (minimal surface) to +1.0 (convex, blue), (*c*) curvedness, mapped from −4.0 (flat, green) through 0.0 (unit sphere) to +0.4 (singular, blue), and (*d*) 2D fingerprint plots with *d*_e_ and *d*_i_ ranging from 0.6 to 2.8 Å.

**Figure 12:**
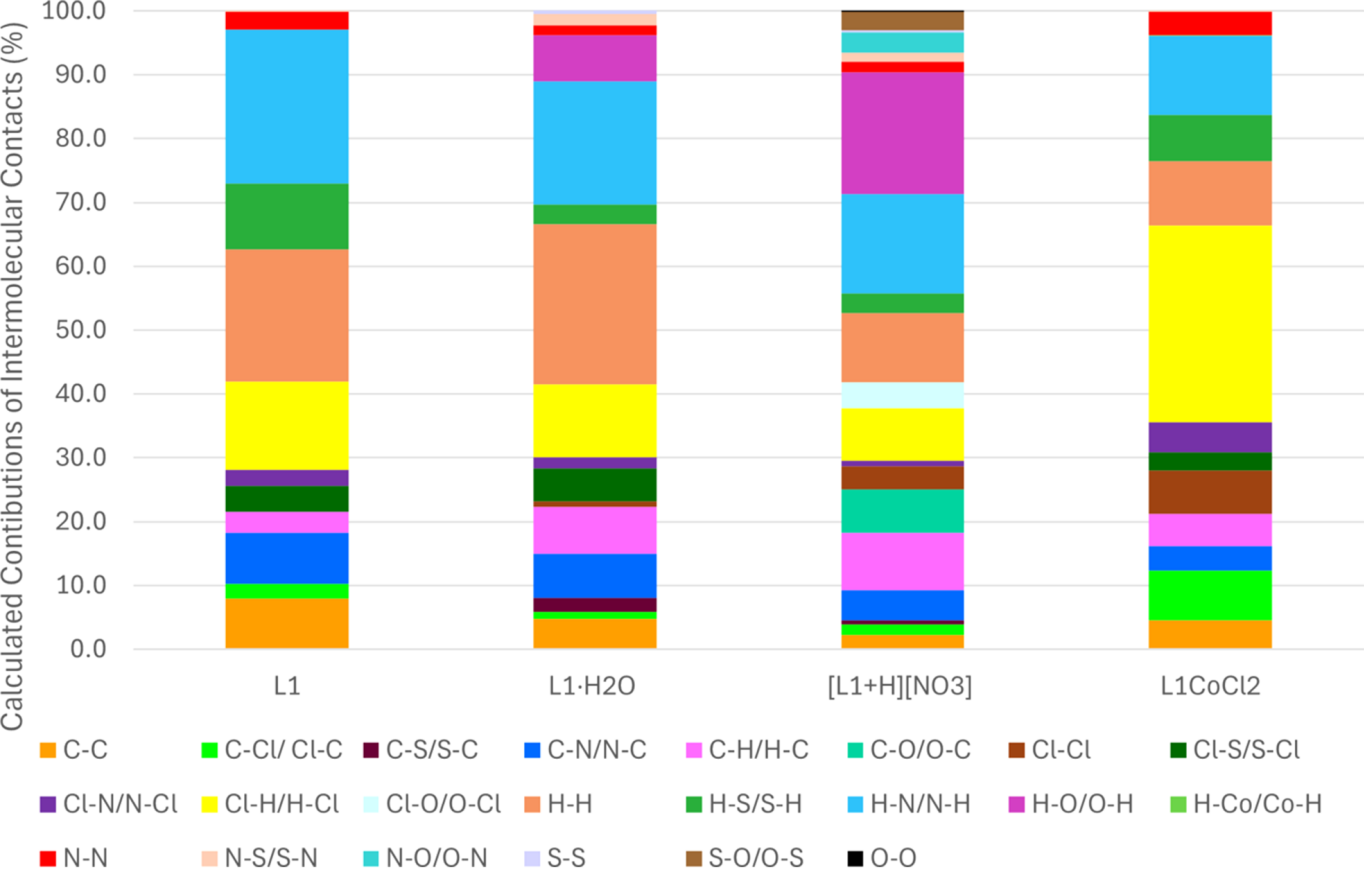
The calculated contributions of all the inter­molecular contacts.

**Figure 13:**
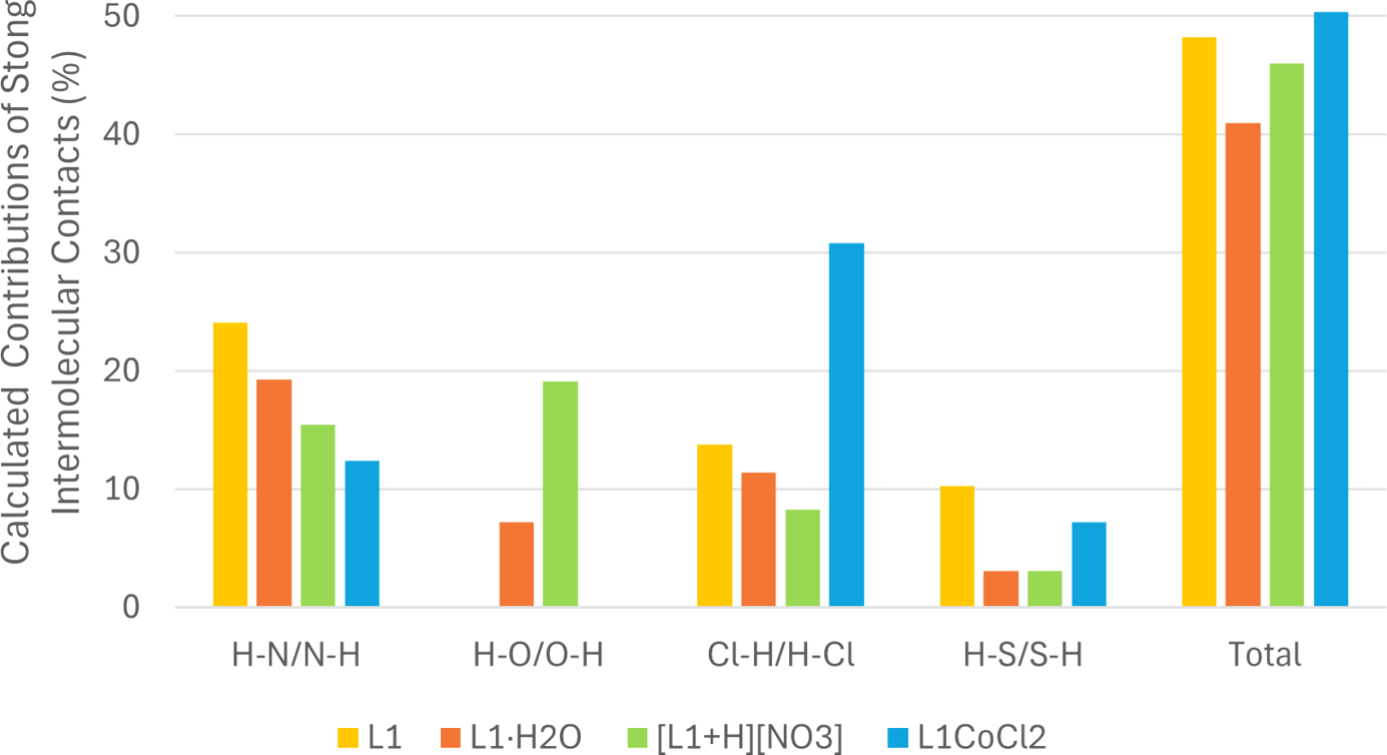
The calculated contributions of the strong inter­molecular contacts.

It is worth noting that in both conformations, the ligand adopts a planar geometry, which is consistent with the pres­ence of π–π (C⋯C) inter­actions, as revealed by the Hirshfeld surface analysis. When curvedness is mapped, flat green regions are observed at the sites of π–π stacking [Fig. 11 ▸(*c*)]. Similarly, when shape index is mapped, adjacent red and blue triangles appear in these regions, indicating the location of these inter­actions [Fig. 11 ▸(*b*)]. The C⋯C contributions to the total inter­molecular contacts are greater in **L1** and **L1·H_2_O** than in the nitrate salt, **[L1+H][NO_3_]** (Fig. 12 ▸; also Figs. S6–S9 and Table S1 in the supporting information). While all metal-free structures exhibit planarity (Figs. S2–S5), the nitrate salt appears more planar than the water solvate, despite the lower C⋯C contributions. This increased planarity likely correlates with enhanced H⋯O inter­actions (Fig. 12 ▸). In Fig. 13 ▸, strong hy­dro­gen-bond inter­actions are defined as H⋯N/N⋯H, H⋯O/O⋯H, Cl⋯H/H⋯Cl and H⋯S/S⋯H. Not all inter­action types are relevant for every structure (*e.g.***L1** does not contain any O atoms).

## Conclusion

Herein, we have reported the dissymmetric thio­ether *N*,*N*′-bi­dentate ligand **L1**, which exhibits conformational flexibility and variable hy­dro­gen-bonding behaviour across a series of structurally characterized com­pounds, including its neutral form, water solvate, protonated nitrate salt and a cobalt(II) com­plex. In the uncoordinated and unprotonated forms, the hy­dro­gen-bond capacity of **L1** is unfulfilled. Upon protonation or metal coordination, however, the ligand adopts a conformation that enables full participation of its hy­dro­gen-bond donors, demonstrating the structural adap­ta­bility of **L1** in response to its environment. These insights are foundational to further exploration of **L1** as a platform for mol­ecular recognition and ion sensing.

Future work is planned for ligand elaboration *via* the **L1** chloride substituent, which provides a synthetic handle for further functionalization. These efforts are focused on ex­panding the utility of this ligand family in supra­molecular and coordination-based applications.

## Supplementary Material

Crystal structure: contains datablock(s) l1, l1-h2o, l1h-no3, l1cocl2, global. DOI: 10.1107/S205322962500823X/yd3063sup1.cif

Structure factors: contains datablock(s) l1. DOI: 10.1107/S205322962500823X/yd3063l1sup2.hkl

Structure factors: contains datablock(s) l1-h2o. DOI: 10.1107/S205322962500823X/yd3063l1-h2osup3.hkl

Structure factors: contains datablock(s) l1h-no3. DOI: 10.1107/S205322962500823X/yd3063l1h-no3sup4.hkl

Structure factors: contains datablock(s) l1cocl2. DOI: 10.1107/S205322962500823X/yd3063l1cocl2sup5.hkl

Supporting information file. DOI: 10.1107/S205322962500823X/yd3063l1sup6.cml

Supporting information file. DOI: 10.1107/S205322962500823X/yd3063l1-h2osup7.cml

Supporting information file. DOI: 10.1107/S205322962500823X/yd3063l1h-no3sup8.cml

Additional figures and tables. DOI: 10.1107/S205322962500823X/yd3063sup9.pdf

CCDC references: 2472058, 2472057, 2472056, 2472055

## Figures and Tables

**Table 1:** Experimental details Experiments were carried out at 110 K with Mo *K*α radiation using a Bruker APEXII CCD diffractometer. H atoms were treated by a mixture of independent and constrained refinement.

	**L1**	**L1·H_2_O**	**[L1+H][NO_3_]**	**L1CoCl_2_**
Crystal data				
Chemical formula	C_8_H_7_ClN_6_S	C_8_H_7_ClN_6_S·H_2_O	C_8_H_8_ClN_6_S^+^·NO_3_^−^	[CoCl_2_(C_8_H_7_ClN_6_S)]
*M*_r_	254.71	272.72	317.72	384.54
Crystal system, space group	Orthorhombic, *Pna*2_1_	Monoclinic, *P*2_1_/*n*	Monoclinic, *P*2_1_/*c*	Monoclinic, *P*2_1_/*c*
*a*, *b*, *c* (Å)	13.5864 (6), 6.8262 (3), 11.6234 (5)	3.8868 (15), 14.652 (6), 19.154 (7)	19.541 (8), 18.048 (9), 6.851 (4)	7.763 (2), 24.510 (9), 7.152 (3)
α, β, γ (°)	90, 90, 90	90, 90.147 (7), 90	90, 92.765 (11), 90	90, 97.464 (4), 90
*V* (Å^3^)	1077.99 (8)	1090.8 (8)	2413 (2)	1349.4 (9)
*Z*	4	4	8	4
μ (mm^−1^)	0.53	0.54	0.51	2.01
Crystal size (mm)	0.19 × 0.17 × 0.08	0.28 × 0.13 × 0.13	0.25 × 0.07 × 0.06	0.39 × 0.16 × 0.05

Data collection				
Absorption correction	Multi-scan (*SADABS2016*; Bruker, 2016 ▸)	Multi-scan (*SADABS2016*; Bruker, 2016 ▸)	Multi-scan (*SADABS2016*; Bruker, 2016 ▸)	Empirical (using intensity measurements) (*TWINABS2012*; Bruker, 2012 ▸)
*T*_min_, *T*_max_	0.688, 0.747	0.685, 0.749	0.674, 0.746	0.532, 0.745
No. of measured, independent and observed [*I* > 2σ(*I*)] reflections	87768, 5788, 5131	128142, 8646, 6849	90727, 6784, 4861	71073, 2659, 2324
*R*_int_	0.057	0.051	0.090	0.067
(sin θ/λ)_max_ (Å^−1^)	0.863	0.981	0.696	0.618

Refinement				
*R*[*F*^2^ > 2σ(*F*^2^)], *wR*(*F*^2^), *S*	0.031, 0.075, 1.04	0.030, 0.088, 1.04	0.043, 0.105, 1.03	0.044, 0.087, 1.15
No. of reflections	5788	8646	6784	2659
No. of parameters	161	178	399	184
No. of restraints	7	7	44	6
Δρ_max_, Δρ_min_ (e Å^−3^)	0.41, −0.26	0.64, −0.36	0.88, −0.92	0.69, −0.43
Absolute structure	Flack *x* determined using 2256 quotients [(*I*^+^) − (*I*^−^)]/[(*I*^+^) + (*I*^−^)] (Parsons *et al.*, 2013 ▸)	–	–	–
Absolute structure parameter	0.02 (2)	–	–	–

Computer programs: *APEX2* (Bruker, 2018 ▸), *SAINT* (Bruker, 2018 ▸), *SHELXT2018* (Sheldrick, 2015*a* ▸), *SHELXL2019* (Sheldrick, 2015*b* ▸) and *OLEX2* (Dolomanov *et al.*, 2009 ▸).

**Table 2:** Selected hy­dro­gen bonds (Å, °) described by primary associated graph-set notation

*D*	H	*A*	*D*—H	H⋯*A*	*D*⋯*A*	*D*—H⋯*A*	Associated graph-set motif
**L1**							
N5	H5*B*	N4^i^	0.84 (2)	2.21 (2)	3.023 (2)	162 (3)	*C*(10)
N6	H6*A*	N1^ii^	0.81 (2)	2.29 (2)	3.0215 (19)	150 (2)	*C*(6)
N6	H6*B*	N3^iii^	0.853 (19)	2.33 (2)	3.087 (2)	148 (2)	*C*(8)
							
**L1·H_2_O**							
N6	H6*A*	O1^iv^	0.873 (11)	2.191 (11)	3.0625 (13)	176.3 (13)	*D*(2)
N6	H6*B*	O1^v^	0.854 (11)	2.217 (11)	3.0472 (12)	164.2 (13)	*D*(2)
N5	H5*A*	N4^vi^	0.855 (11)	2.588 (12)	3.3899 (12)	156.7 (12)	*C*(10)
N5	H5*B*	N1^vii^	0.848 (11)	2.282 (12)	3.1182 (14)	169.0 (13)	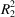 (8)
							
**[L1+H][NO_3_]**							
N1	H1	N3	0.88 (2)	1.93 (3)	2.681 (3)	142 (3)	*S*(6)
N5	H5*A*	N4^viii^	0.837 (16)	2.319 (17)	3.134 (3)	165 (2)	*C*(10)
N5	H5*B*	O1	0.834 (16)	1.966 (17)	2.789 (3)	169 (3)	*D*(2)
N6	H6*A*	O3^viii^	0.844 (16)	2.000 (16)	2.843 (3)	176 (3)	*D*(2)
N6	H6*B*	N8	0.844 (16)	2.142 (17)	2.983 (3)	174 (3)	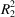 (8)
N7	H7	N9	0.89 (2)	1.92 (3)	2.675 (3)	142 (3)	*S*(6)
N11	H11*A*	O4	0.838 (16)	2.112 (18)	2.936 (4)	167 (3)	*D*(2)
N11	H11*A*	O5*A*	0.838 (16)	1.807 (19)	2.630 (8)	167 (3)	*D*(2)
N11	H11*B*	N10^ix^	0.845 (16)	2.328 (18)	3.150 (3)	164 (3)	*D*(2)
N12	H12*A*	N2	0.841 (15)	2.203 (16)	3.040 (3)	174 (2)	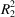 (8)
N12	H12*B*	O6^ix^	0.842 (16)	2.10 (2)	2.872 (4)	152 (3)	*D*(2)
N12	H12*B*	O4*A*^ix^	0.842 (16)	1.922 (18)	2.763 (6)	175 (3)	*D*(2)
							
**L1CoCl_2_**							
N5	H5*A*	Cl2^*x*^	0.81 (3)	2.63 (3)	3.385 (4)	156 (5)	*C*(10)
N5	H5*B*	Cl3	0.81 (3)	2.53 (3)	3.320 (4)	163 (5)	*S*(6)
N6	H6*A*	Cl2^xi^	0.81 (3)	2.61 (3)	3.400 (4)	164 (5)	*C*(10)
N6	H6*B*	N2^xii^	0.81 (3)	2.29 (3)	3.096 (5)	174 (5)	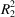 (8)

Symmetry codes: (i) −*x* + 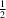, *y* + 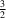, *z* + 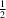; (ii) *x* + 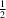, −*y* + 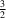, *z*; (iii) *x* + 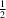, −*y* + 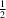, *z*; (iv) *x* + 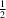, −*y* + 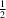, *z* − 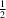; (v) −*x* + 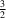, *y* − 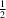, −*z* + 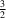; (vi) *x* + 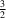, −*y* + 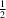, *z* − 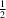; (vii) −*x* + 2, −*y* + 1, −*z* + 1; (viii) −*x* + 1, *y* + 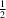, −*z* + 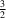; (ix) −*x* + 2, *y* − 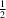, −*z* + 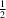; (x) *x*, *y*, *z* + 1; (xi) −*x* + 1, −*y* + 1, −*z* + 2; (xii) −*x* + 2, −*y* + 1, −*z* + 2.
