# Supplementary material for: Structural adaptability and hy­dro­gen bonding in a dissymmetric pyrimidine thio­ether ligand
Source: Acta Crystallogr C Struct Chem. 2025 Oct 6;81(Pt 11):598–606. doi: 10.1107/S205322962500823X (PMC12587320; doi:10.1107/S205322962500823X)
Supplement: Supplementary file 9 [file c-81-00598-sup9.pdf]

STRUCTURAL
CHEMISTRY

Volume 81 (2025)

Supporting information for article:

Structural adaptability and hydrogen bonding in a dissymmetric pyrimidine thioether ligand

Kaycee Anoliefo, Kaitlyn Brown, Lana K. Hiscock, Paul D. Boyle and Louise N. Dawe

Table S1 Percentage of the total surface area of close intermolecular contacts between atoms inside and outside the surface.

Inside/ Outside Contacts	L1 (%)	L1-H_2O (%)	L1-NO_3 (%)	L1-$CoCl_2$ (%)
C-C	8.0	4.8	2.2	4.5
C-Cl/ Cl-C	2.2	1.1	1.7	7.8
C-S/S-C		2.2	0.7	
C-N/N-C	8.0	6.9	4.7	3.9
C-H/H-C	3.3	7.3	8.9	5.0
C-O/O-C			6.9	
Cl-Cl		0.9	3.6	6.8
Cl-S/S-Cl	4.1	5.1		2.9
Cl-N/N-Cl	2.5	1.8	0.8	4.7
Cl-H/H-Cl	13.8	11.4	8.3	30.8
Cl-O/O-Cl		>0.0	4.0	
H-H	20.8	25.1	10.9	10.1
H-S/S-H	10.3	3.1	3.1	7.2
H-N/N-H	24.1	19.3	15.5	12.4
H-O/O-H		7.2	19.1	
H-Co/Co-H				0.1
N-N	2.7	1.5	1.6	3.6
N-S/S-N	0.2	1.8	1.5	0.3
N-O/O-N			3.1	
N-Co/Co-N				>0.0
S-S		0.6	0.4	
S-O/O-S			2.8	

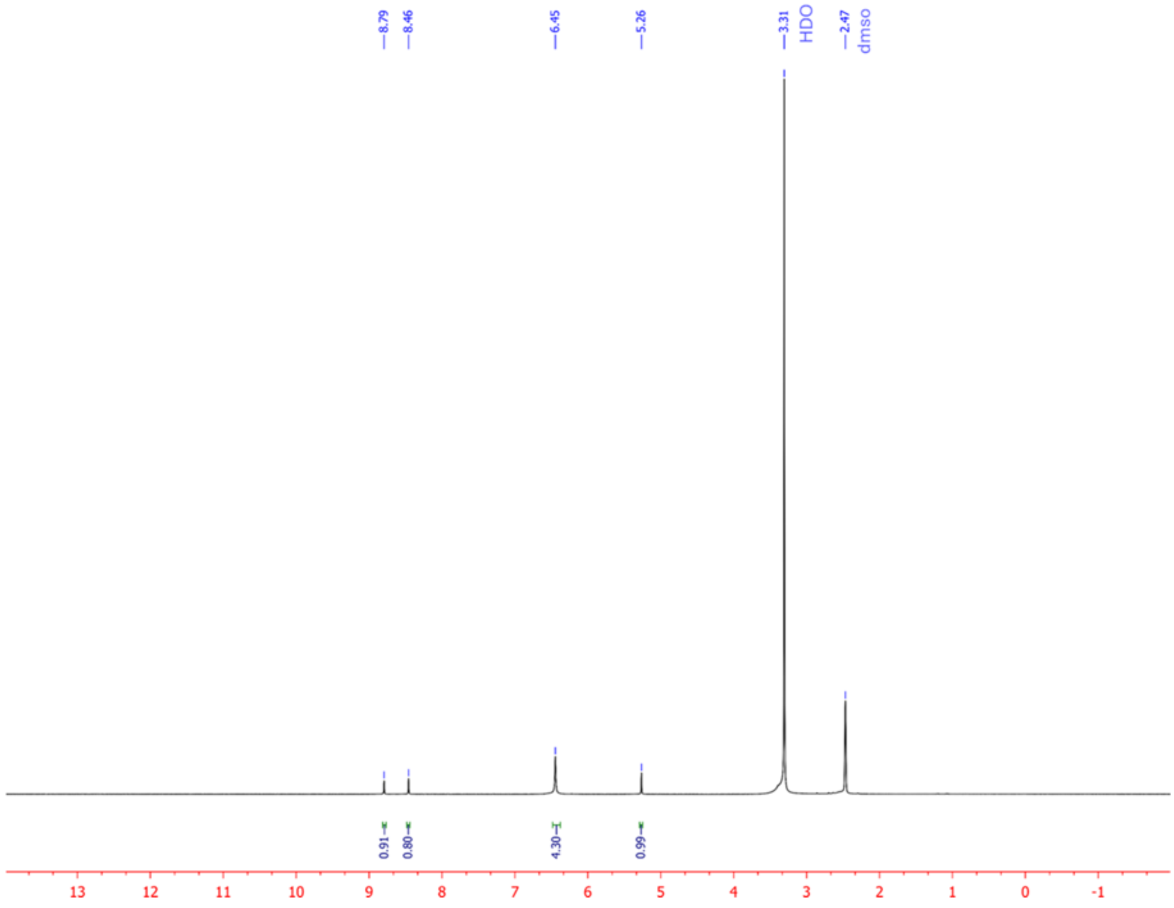

Figure S1 ^{1}H NMR for **L1** (400 MHz, DMSO-d_6) δ: 8.79 (s, 1H), 8.46 (s, 1H), 6.45 (s, 4H), 5.26 (s, 1H).

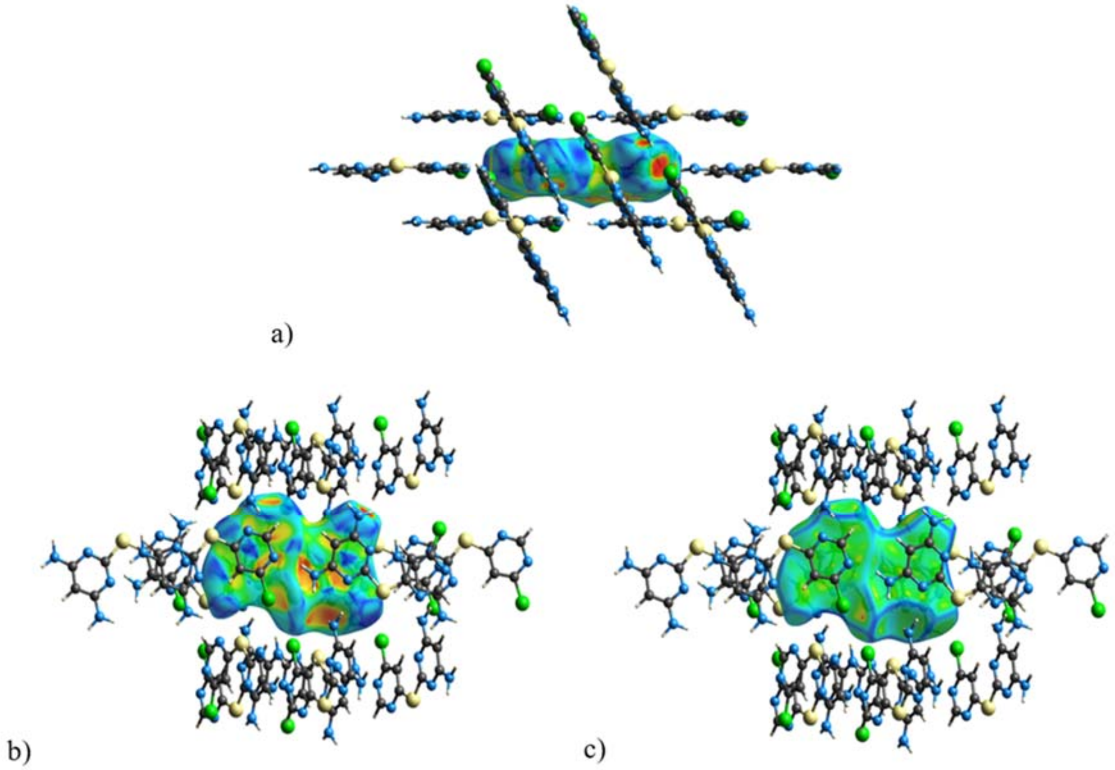

Figure S2 Planar Stacking of **L1** (a) when shape index is mapped (b) and when curvedness is mapped (c). Showing atoms within a 3.80 Å radius of all the atoms on **L1.**

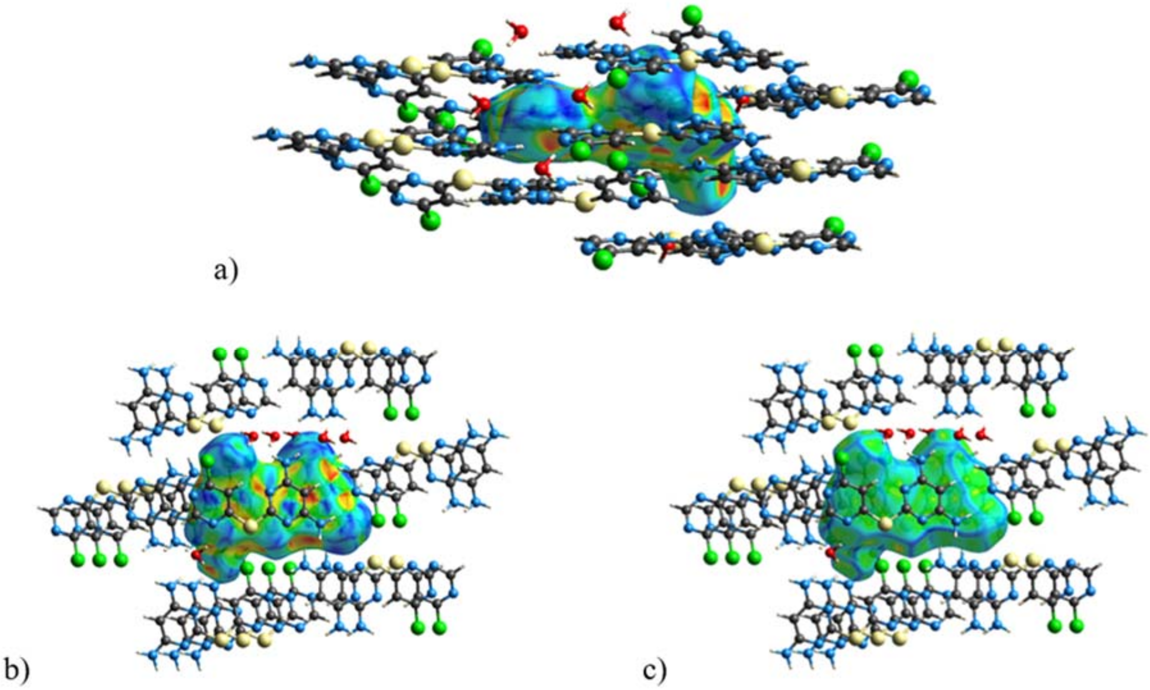

Figure S3 Planar Stacking of **L1 H_2O** (a) when shape index is mapped (b) and when curvedness is mapped (c). Showing atoms within a 3.80 Å radius of all the atoms on **L1 H_2O.**

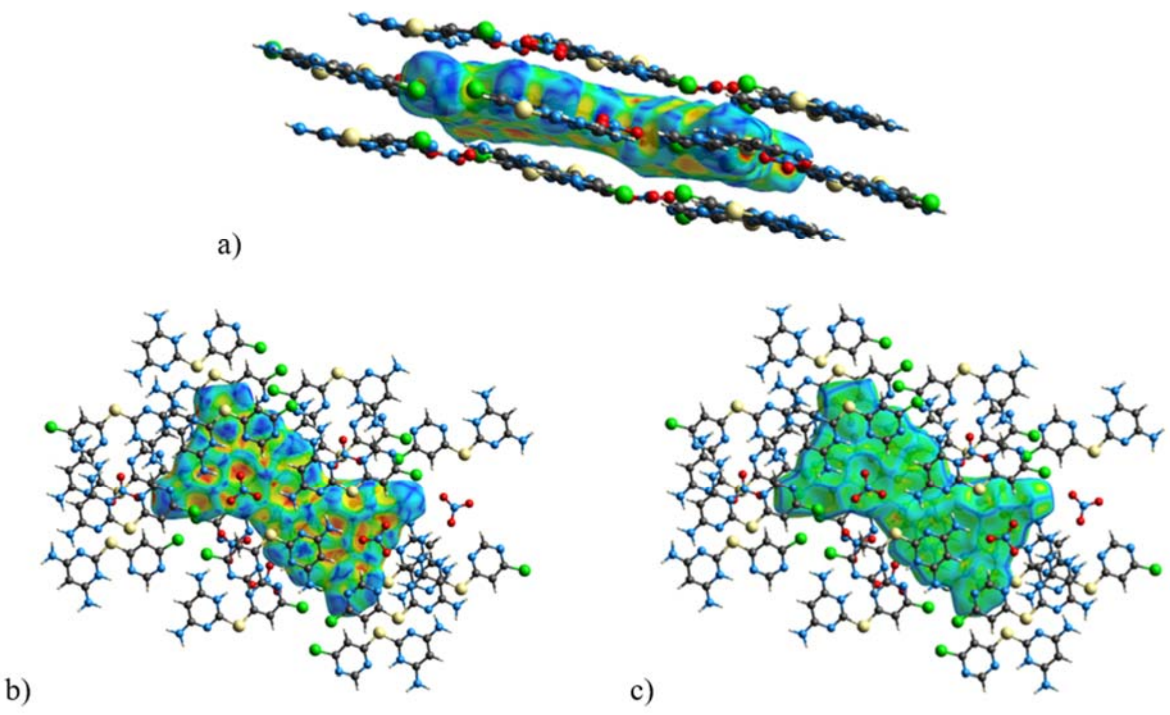

Figure S4 Planar Stacking of **[L1+H][NO_3]** (a) when shape index is mapped (b) and when curvedness is mapped (c). Showing atoms within a 3.80 Å radius of all the atoms on **[L1+H][NO_3]**.

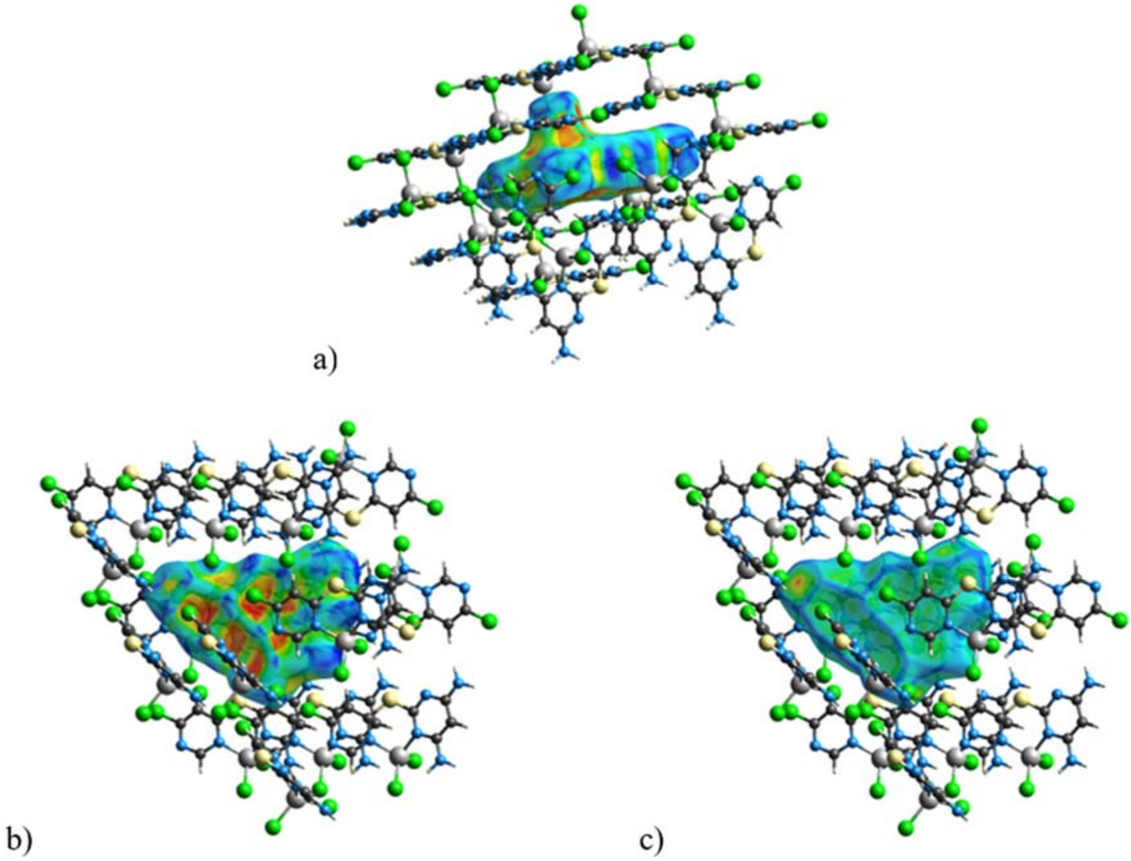

Figure S5 Planar Stacking of **L1CoCl$_2$** (a) when shape index is mapped (b) and when curvedness is mapped (c). Showing atoms within a 3.80 Å radius of all the atoms on **L1CoCl$_2$.**

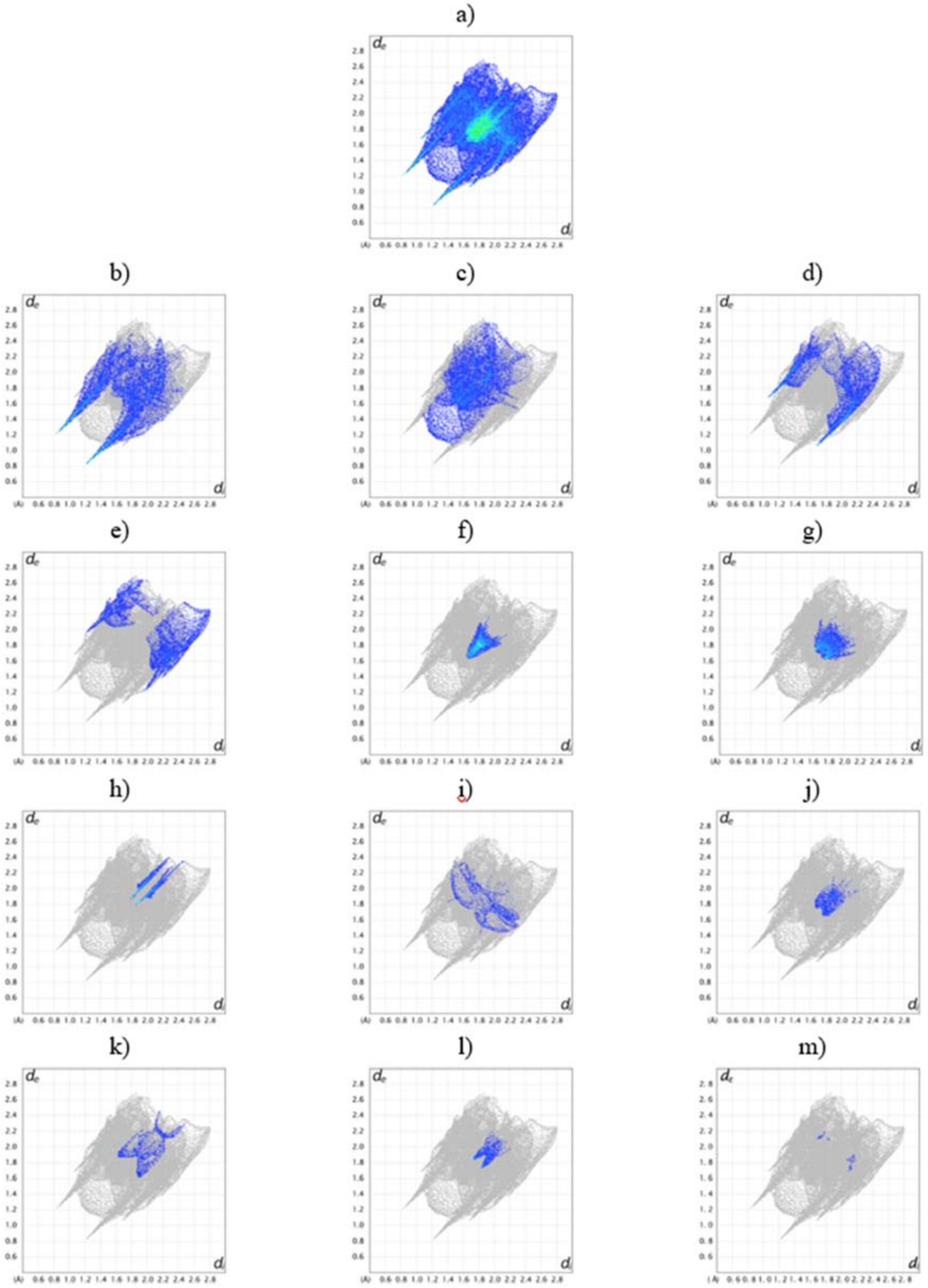

Figure S6 2D finger print plots for **L1**, a) All interactions:100 % ; b) N-H/H-N interactions: 24.1 % ; c) H-H interactions: 20.8 % ; d) Cl-H/H-Cl interactions: 13.8 % ; e) S-H/H-S interactions: 10.3 % ; f) C-C interactions: 8.0 % ; g) N-C/C-N interactions: 8.0% ; h) Cl-S/S-Cl interactions: 4.1 % ; i) H-

C/C-H interactions: 3.3 % ; j) N-N interactions: 2.7 % ; k) Cl-N/N-Cl interactions: 2.5 % ; l) Cl-C/C-Cl interactions: 2.2 % ; m) S-N/N-S interactions: 0.2 %.

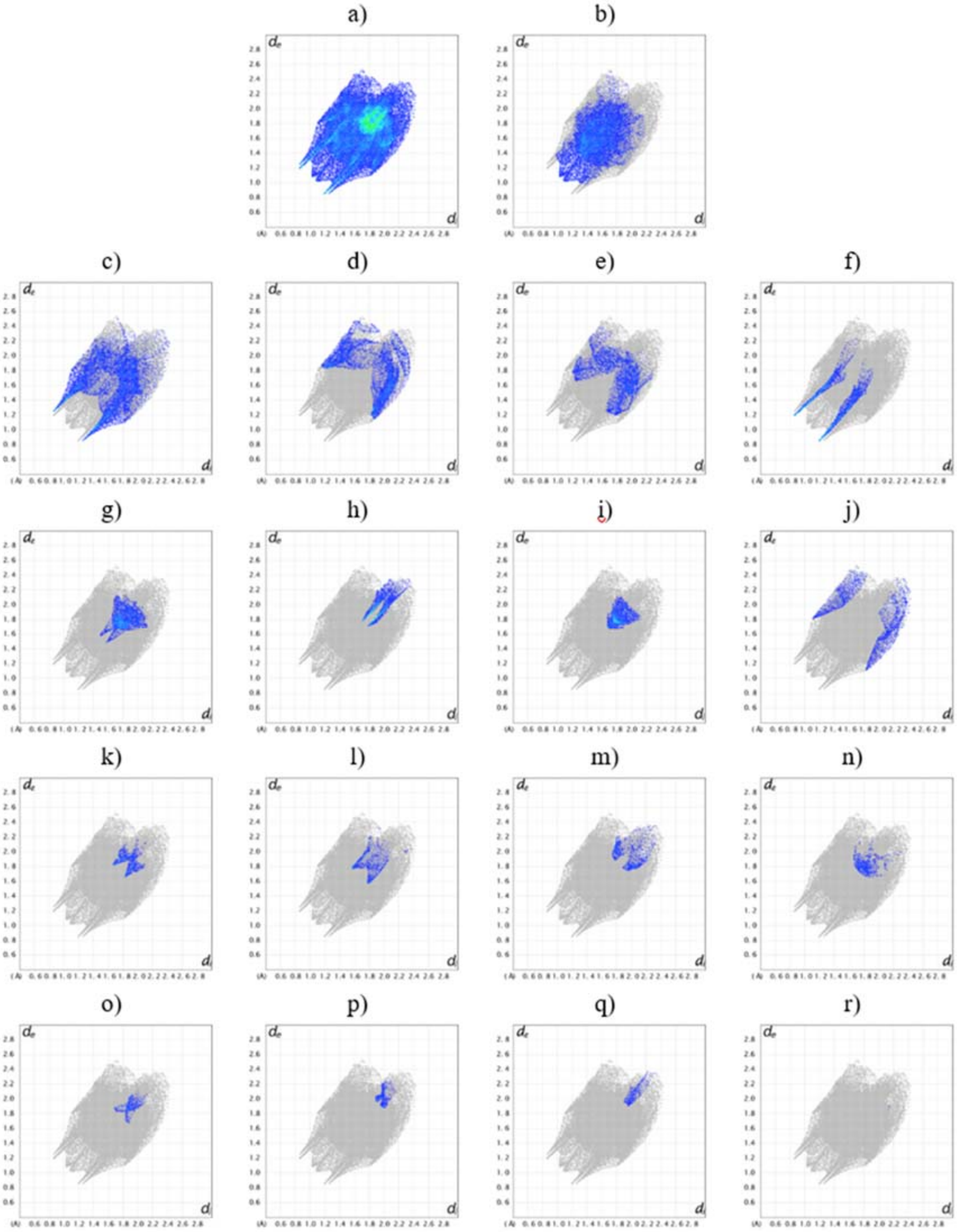

Figure S7 2D finger print plot for **L1 H_2O**, a) All interactions: 100 % ; b) H-H interactions: 25.1 % ; c) N-H/H-N interactions: 19.3 % ; d) Cl-H/H-Cl interactions: 11.4 % ; e) C-H/H-C interactions: 7.3 % ; f) H-O/O-H interactions: 7.2 % ; g) C-N/N-C interactions: 6.9 % ; h) Cl-S/S-Cl interactions: 5.1 % ; i) C-C interactions: 4.8 % ; j) S-H/H-S interactions: 3.1 % ; k) S-C/C-S interactions: 2.2 % ; l) Cl-N/N-Cl interactions:1.8 % ; m) S-N/N-S interactions: 1.8 % ; n) N-N interactions: 1.5 % ; o) Cl-C/C-

Cl interactions: 1.1 % ; p) Cl-Cl interactions: 0.9 % ; q) S-S interactions: 0.6 % ; r) Cl-O/O-Cl interactions: >0.0 %.

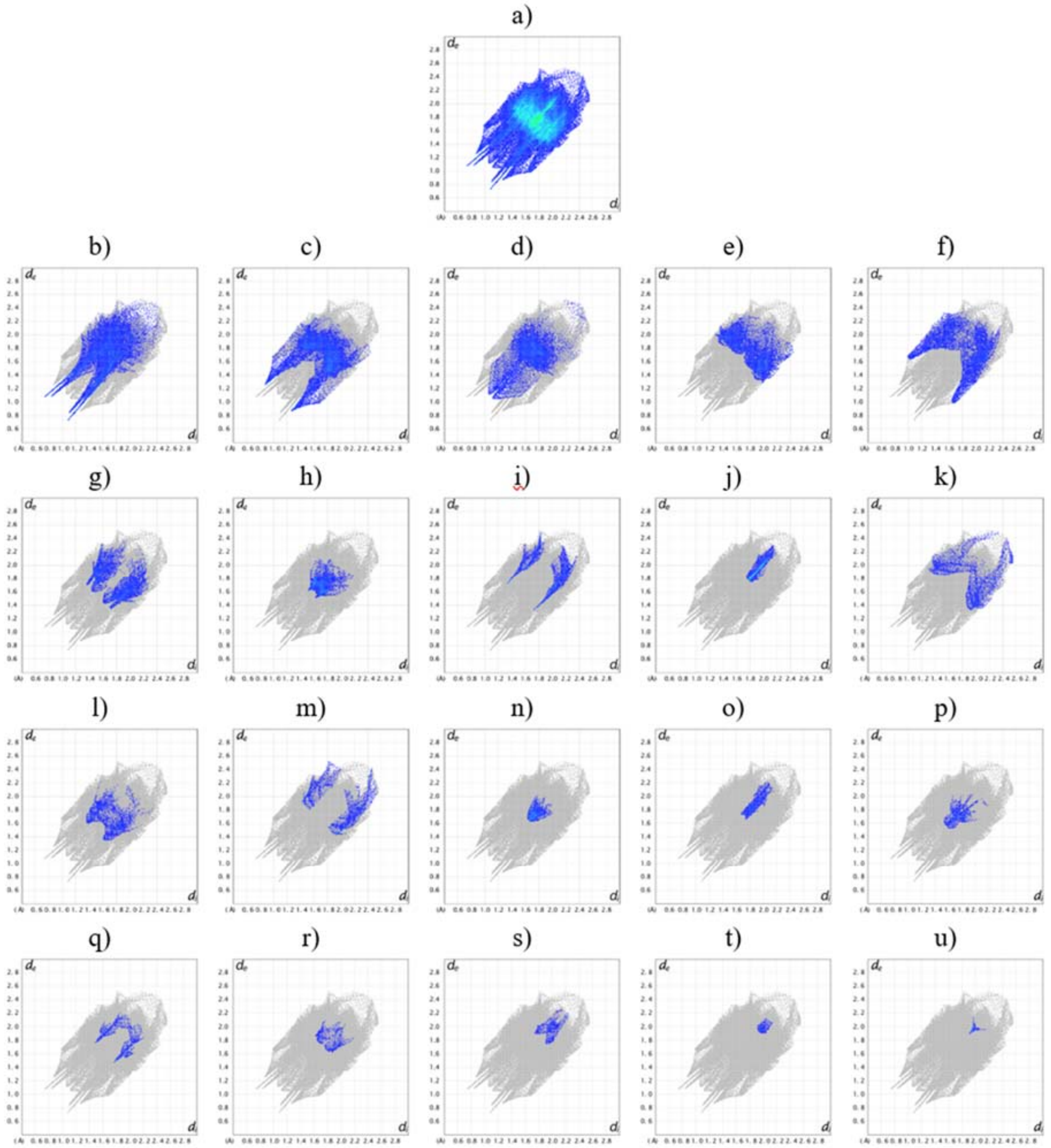

Figure S8 2D finger print plot for **[L1+H][NO$_3$]**, a) All interactions: 100 % ; b) H-O/O-H interactions: 19.1 % ; c) N-H/H-N interactions: 15.5 % ; c) H-H interactions: 10.9 % ; e) H-C/C-H interactions: 8.9 % ; f) Cl-H/H-Cl interactions: 8.3 % ; g) C-O/O-C interactions: 6.9 % ; h) N-C/C-N interactions: 4.7 % ; i) Cl-O/O-Cl interactions: 4.0 % ; j) Cl-Cl interactions: 3.6 % ; k) H-S/S-H interactions: 3.1 % ; l) N-O/O-N interactions: 3.1 % ; m) S-O/O-S interactions: 2.8 % ; n) C-C interactions: 2.2 % ; o) Cl-C/C-Cl interactions: 1.7 % ; p) N-N interactions: 1.6 % ; q) N-S/S-N interactions: 1.5 % ; r) Cl-N/N-Cl interactions: 0.8 % ; s) C-S/S-C interactions: 0.7 % ; t) S-S interactions: 0.4 % ; u) O-O interactions: 0.3 %.

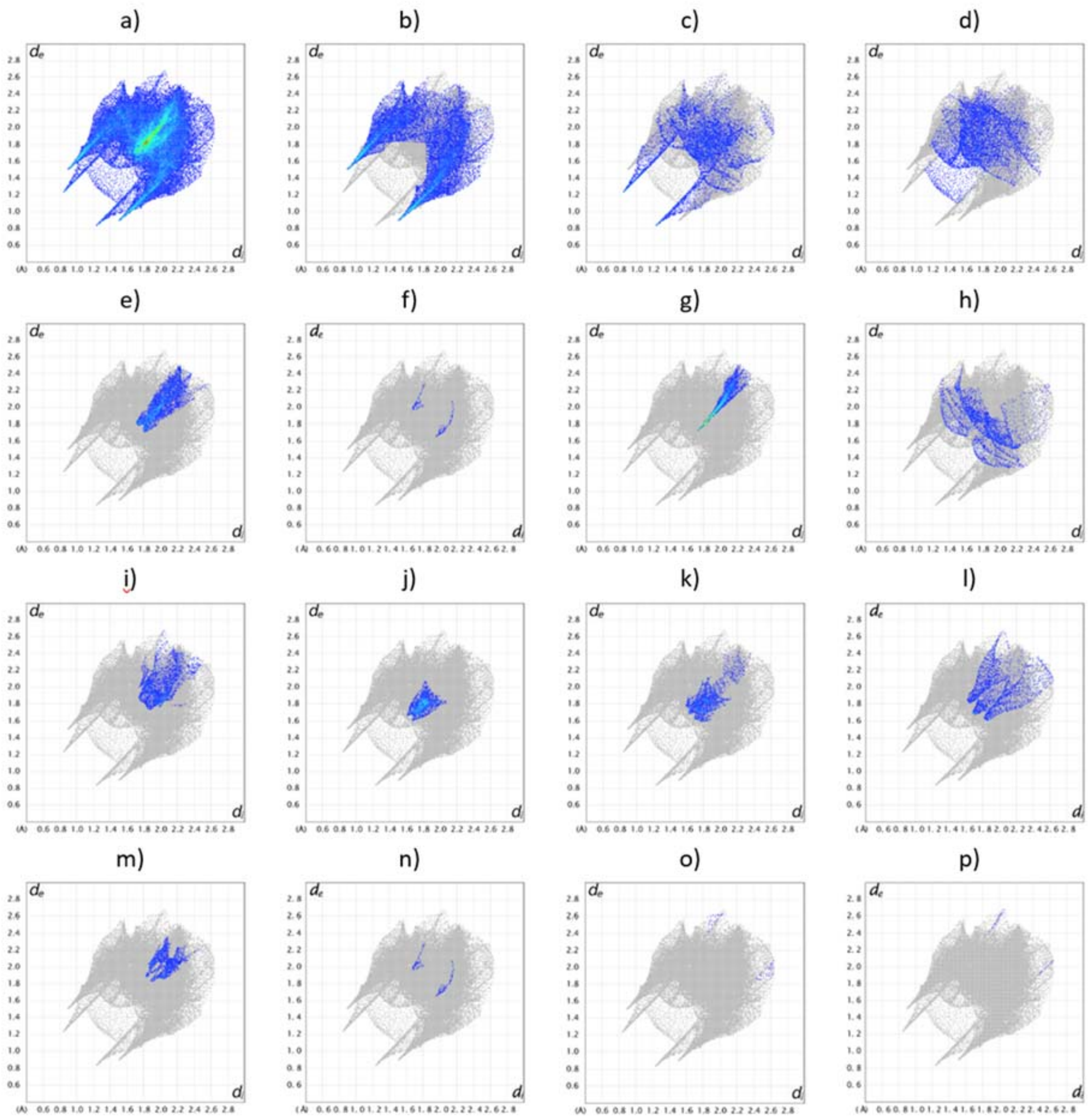

Figure S9 2D finger print plot for **L1CoCl₂**, a) All interactions: 100 % ; b) Cl-H/H-Cl interactions: 30.8 % ; c) N-H/H-N interactions: 12.4 % ; d) H-H interactions: 10.1 % ; e) Cl-C/C-Cl interactions: 7.8 % ; f) S-H/H-S interaction: 7.2 % ; g) Cl-Cl interactions: 6.8 % ; h) C-H/ H-C interaction: 5.0 % ; i) Cl-N/N-Cl interactions: 4.7 % ; j) C-C interactions: 4.5 % ; k) C-N/N-C interactions: 3.9 % ; l) N-N interactions: 3.6 % ; m) Cl-S/S-Cl interactions: 2.9 % ; n) S-N/N-S interactions: 0.3 % ; o) Co-H/H-Co interactions: 0.1 % ; p) N-Co/Co-N interactions: >0.0 %.
